# The Dual Facets of Emotion Perception in Adult Attachment Representations: A Systematic Review on Impathy and Empathy

**DOI:** 10.3390/brainsci16060651

**Published:** 2026-06-19

**Authors:** Dirk W. Eilert, Philipp Mensah, Anna Buchheim

**Affiliations:** 1 Institute of Psychology, University of Innsbruck, 6020 Innsbruck, Austria; anna.buchheim@uibk.ac.at; 2 Emotion Processing Lab, Eilert-Academy, 13585 Berlin, Germany; philipp.mensah@eilert-akademie.de

**Keywords:** attachment representation, adult attachment interview, adult projective picture system, reflective functioning, mentalization, theory of mind, emotion processing, emotion perception, impathy, empathy

## Abstract

**Highlights:**

**What are the main findings?**

- Across 38 studies, attachment representations were systematically associated with intrapersonal and interpersonal emotion perception, with the strongest evidence for reflective functioning, the Impathy dimensions Perceiving and Understanding, and cognitive-empathic processes.
- A meta-analysis of eight studies showed a large positive association between secure attachment representations and reflective functioning (*r* = 0.64, 95% CI [0.50, 0.74]).

**What are the implications of the main findings?**

- The findings support the assumption that attachment-related differences in emotion perception may represent an important mechanism underlying intergenerational attachment transmission.
- Intrapersonal and interpersonal emotion perception may represent important attachment-sensitive targets for psychotherapy, prevention, and emotion-focused interventions.

**Abstract:**

**Background/Objectives:** Emotion processing has increasingly been conceptualized as a transdiagnostic mechanism underlying psychological adaptation and psychopathology. From an attachment perspective, individual differences in emotion perception may be rooted in internal working models shaped by early relationships. This systematic review synthesized the literature on the relationship between adult attachment representations and intrapersonal emotion perception (Impathy) and interpersonal emotion perception (Empathy). **Methods:** The review was conducted in accordance with Preferred Reporting Items for Systematic Reviews and Meta-Analyses (PRISMA) guidelines. A systematic search was conducted on 9 February 2026, in PsycINFO, PsycArticles, and PubMed. Studies were included if they investigated adolescents or adults, assessed attachment representations using narrative-based measures (Adult Attachment Interview (AAI) or Adult Attachment Projective Picture System (AAP)), and examined intrapersonal and/or interpersonal emotion perception. Findings were synthesized narratively, and a random-effects meta-analysis examined the association between attachment security and reflective functioning. **Results:** Thirty-eight studies, including 2736 participants, met the inclusion criteria. Across studies, attachment representations were systematically associated with intrapersonal and interpersonal emotion perception. The strongest evidence emerged for reflective functioning, the Impathy dimensions Perceiving and Understanding, and cognitive-empathic processes. Secure attachment representations were consistently associated with higher reflective functioning and more adaptive emotion perception, whereas insecure and especially unresolved attachment representations were linked to impairments in emotional self-awareness, alexithymia-related processes, differentiated emotional understanding, and cognitive-empathic processing. The meta-analysis showed a large positive association between secure attachment representations and reflective functioning (*k* = 8; *r* = 0.64, 95% CI [0.50, 0.74]). **Conclusions:** Attachment representations appear systematically associated with the perceptual foundations of emotion processing. Intrapersonal and interpersonal emotion perception may therefore represent attachment-sensitive processes relevant to psychological adaptation, psychopathology, caregiving, and therapeutic change.

## 1. Introduction

### 1.1. Emotion Processing as a Transdiagnostic Core Mechanism

Emotions are central to adaptive functioning, shaping perception, cognition, and behavior across contexts [1,2,3,4]. Beyond their immediate regulatory and motivational functions, a growing body of research suggests that disturbances in emotion processing represent a transdiagnostic mechanism underlying a broad range of mental disorders. Rather than being confined to specific diagnostic categories, impairments in the way individuals perceive, regulate, and express emotions appear to constitute a general vulnerability dimension that cuts across traditional disorder boundaries (e.g., [5,6,7,8]).

This perspective is consistent with contemporary models of psychopathology emphasizing shared underlying liabilities, such as the general factor of psychopathology (p-factor; [9]). Within this framework, deficits in emotion processing have been proposed as a key explanatory mechanism for both symptom co-occurrence and heterogeneity across disorders. Empirical evidence indicates that alterations in emotion processing are evident in mood disorders, anxiety disorders, and personality disorders alike, supporting the notion that they reflect a core dimension of socio-emotional functioning rather than disorder-specific impairments (e.g., [10,11,12,13]).

### 1.2. Attachment as a Foundation of Emotion Processing

From an attachment-theoretical perspective, individual differences in emotion processing are rooted in early relational experiences. According to Bowlby, the development of the self is fundamentally embedded in emotional exchanges with primary caregivers, giving rise to internal working models that organize expectations, perceptions, and responses in emotionally salient contexts [14,15,16,17].

Bowlby highlighted the crucial role of early, pre-verbal social experiences in shaping the internal working model of attachment ([17], p. 157): “During the earliest years of our lives, indeed, emotional expression and its reception are the only means of communication we have, so that the foundations of our working models of self and attachment figure are perforce laid using information from that source alone.” These internal working models are thought to shape multiple components of emotion processing across the lifespan, influencing how individuals perceive and interpret emotions in themselves and others, regulate emotional experiences, and express emotional states. Importantly, these processes operate largely outside of conscious awareness, highlighting the importance of assessing attachment at the representational level (e.g., Adult Attachment Interview, Adult Attachment Projective Picture System) rather than relying solely on self-report measures [14,18,19,20,21].

Beyond their role in shaping specific emotional processes, attachment representations have also been identified as transdiagnostic risk factors for psychopathology. Recent neurobiological evidence suggests that unresolved attachment, particularly in the context of loss or trauma, is associated with structural alterations in brain systems implicated in emotional and cognitive integration, independent of general psychopathology severity [22,23]. Specifically, while a general psychopathology factor has been linked to reduced white matter integrity in interhemispheric pathways, unresolved attachment representations show distinct associations with alterations in tracts related to the integration of emotional and perceptual information.

These findings underscore that attachment-related processes do not merely reflect downstream consequences of psychopathology but constitute an independent dimension of vulnerability, likely exerting their influence through fundamental mechanisms of emotion processing.

### 1.3. Toward a Multidimensional Conceptualization of Emotion Processing

Although emotion regulation has received considerable attention in both attachment research and clinical psychology, it represents only one component of a broader system of emotion processing. A closer look at the literature reveals that different aspects of emotional functioning have been investigated extensively, yet largely in isolation from one another. One prominent line of research has focused on *emotion regulation*, examining how individuals influence the occurrence, intensity, and duration of emotional responses (e.g., [24,25,26]). A second line has addressed *emotion expression*, particularly the communication of emotional states through facial, vocal, and bodily channels (e.g., [27,28,29,30,31]). In parallel, extensive work on *empathy* has investigated the perception and understanding of others’ emotional states, including both affective and cognitive components (e.g., [32,33,34]). Finally, a growing body of research has examined *self-related aspects of emotion perception*, focusing on processes such as interoceptive awareness, emotional self-awareness, and emotional granularity, which reflect the ability to perceive, identify, and differentiate one’s own emotional states (e.g., [35,36,37,38]).

While each of these research traditions has yielded important insights, they have largely evolved in parallel, with limited theoretical integration across domains. As a result, emotion processing is often conceptualized in terms of isolated components rather than as a coordinated and dynamically interacting system.

To address this fragmentation, the *C-FEP model (Core Functions of Emotion Processing)* provides an integrative framework that differentiates four core functions of emotion processing [39]:

(1) *Intrapersonal emotion perception (impathy)*, referring to the perception and understanding of one’s own emotional states and representing the self-related counterpart to empathy;
(2) *Interpersonal emotion perception (empathy)*, referring to the perception and understanding of others’ emotional states;
(3) *Emotion regulation*, encompassing processes that modulate emotional responses;
(4) *Emotion expression*, referring to the verbal and nonverbal communication of emotions across channels.

This framework integrates previously fragmented lines of research by conceptualizing emotion processing as a coordinated system of four core functions that are functionally distinct yet dynamically interdependent.

Building on this multidimensional perspective, it becomes possible to systematically examine how attachment representations are associated with different core functions of emotion processing. A substantial body of research has already addressed this question with regard to emotion regulation and, to a lesser extent, emotion expression. A recent systematic review by Eilert and Buchheim [40] synthesizing this literature demonstrated consistent associations between attachment representations and regulatory functioning, with secure attachment being linked to greater flexibility and adaptive strategies, whereas insecure and unresolved representations were associated with maladaptive or inflexible patterns of emotion regulation and expression.

In contrast, the perceptual components of emotion processing have received comparatively less systematic attention in attachment research. While individual studies have examined the relationship between attachment and other-related emotion perception (empathy), findings remain heterogeneous and conceptually fragmented. Similarly, emerging research has begun to investigate self-related emotion perception, yet this line of work remains limited and lacks systematic integration. To date, no comprehensive synthesis has examined how attachment representations are associated with both intrapersonal and interpersonal facets of emotion perception within a unified framework.

### 1.4. Intrapersonal Emotion Perception: Impathy

Intrapersonal emotion perception refers to the capacity to perceive, differentiate, and understand one’s own emotional states. Neubrand [41] conceptualized this ability as *Impathy* (introversive empathy), defined as the ability to share in and understand one’s own internal experiences, including emotions, cognitions, and bodily sensations. In contrast to interpersonal emotion perception (empathy), which is directed toward the emotional states of others, impathy focuses on the relationship individuals have with their own inner experiences and therefore represents a central aspect of self-related emotion processing.

According to Neubrand and Gaab [42], impathy comprises four interrelated dimensions: perceiving, meta-position, accepting attitude, and understanding. The first dimension, *perceiving*, describes the ability to direct attention inward and become aware of one’s own emotions, thoughts, and bodily sensations. This dimension is conceptually related to constructs such as interoception, bodily awareness, emotional awareness, self-awareness, and alexithymia, all of which capture differences in the accessibility of internal emotional signals. The second dimension, *meta-position*, refers to the capacity to observe one’s internal experiences without over-identifying with them. It reflects a reflective stance toward one’s emotions and is closely related to constructs such as decentering, self-distancing, cognitive defusion, and metacognitive awareness. The third dimension, *accepting attitude*, describes approaching one’s inner experiences with openness, nonjudgment, and emotional acceptance. Related constructs include mindfulness, self-acceptance, acceptance, and nonjudgmental awareness. Finally, the dimension of *understanding* refers to actively making sense of one’s emotional experiences and integrating them into a coherent understanding of the self. This dimension overlaps conceptually with emotional clarity, emotion differentiation, emotional granularity, self-understanding, and self-knowledge.

Since impathy, as a construct, has only recently been introduced and remains underrepresented in empirical attachment research, studies that explicitly operationalize all four dimensions remain scarce. Therefore, the present review adopts a broader conceptual approach by including related constructs that map onto the four dimensions of impathy. This strategy enables the systematic integration of fragmented research traditions that address different aspects of intrapersonal emotion perception.

### 1.5. Interpersonal Emotion Perception: Empathy

Interpersonal emotion perception refers to the ability to perceive, understand, and emotionally resonate with others’ emotional states. In the present review, empathy is conceptualized as a multidimensional set of capacities that enables individuals to perceive emotional signals in others, mentally understand these experiences, emotionally resonate with them, and maintain self–other differentiation during the empathic process. Consistent with contemporary empathy research, a distinction is made between *cognitive empathy* and *affective empathy*, which represent partially separable yet dynamically interacting dimensions of interpersonal emotion perception (e.g., [32,43,44,45,46]).

*Cognitive empathy* refers to the ability to infer and understand another person’s emotional state, perspective, intentions, or mental experience. It is closely related to constructs such as Theory of Mind (ToM), perspective taking, social cognition, empathic accuracy, emotion recognition, and mentalizing (e.g., [34]). In contrast, *affective empathy* refers to the capacity to emotionally resonate with or share others’ affective states while maintaining awareness of the self-other distinction (e.g., [47]). Related constructs include empathic concern, emotional contagion, emotional responsiveness, and affective sharing.

Importantly, cognitive and affective empathy are not only conceptually distinguishable but are also associated with partially overlapping yet functionally specialized neural systems. Meta-analytic and experimental findings suggest that affective empathy is primarily associated with regions such as the anterior insula (AI), inferior frontal gyrus (IFG), and anterior cingulate cortex (ACC), whereas cognitive empathy and mentalizing more strongly recruit temporoparietal and medial prefrontal regions, including the temporoparietal junction (TPJ), medial prefrontal cortex (mPFC), superior temporal sulcus (STS), and precuneus (e.g., [48,49]). At the same time, evidence suggests dynamic interactions between these systems, arguing against a unitary construct of social understanding and instead supporting the view that interpersonal emotion perception emerges from the flexible interplay of distinct socio-affective and socio-cognitive processes [33].

Although these dimensions are conceptually distinguishable, they are closely interconnected in everyday social functioning. Adaptive interpersonal emotion perception requires not only the cognitive understanding of another person’s emotional experience but also the ability to emotionally resonate without becoming overwhelmed or losing self–other boundaries. Difficulties in either dimension may therefore impair social understanding and interpersonal functioning (e.g., [50,51,52]).

Importantly, empathy research has evolved across multiple partially overlapping research traditions, including ToM, social cognition, emotion recognition, empathic accuracy, and mentalization research. As reflected in the present search strategy, these constructs were considered jointly to capture the broad spectrum of interpersonal emotion perception investigated in attachment research. This broader conceptual approach was necessary because attachment-related studies rarely operationalized empathy within a unified framework; instead, they examined specific facets of interpersonal emotion perception across diverse conceptual and methodological traditions.

### 1.6. Attachment Representations and Emotion Perception

A growing body of research suggests that attachment representations are associated with multiple dimensions of emotion perception [53,54,55,56,57,58,59,60,61,62,63]. Among these, reflective functioning has received particular attention within attachment research.

*Reflective functioning* refers to the ability to understand one’s own and others’ behavior in terms of underlying mental states, including emotions, intentions, and beliefs [64]. As such, reflective functioning inherently integrates self- and other-related emotional understanding and occupies a central position at the intersection of intra- and interpersonal emotion perception. Successful mentalization requires not only awareness of emotional experience but also the ability to maintain a reflective meta-position toward internal states. Previous research demonstrated a positive association between secure attachment and reflective functioning [53], whereas impairments in reflective functioning have been linked to a broad range of psychopathological conditions [65].

Regarding *intrapersonal emotion perception*, several lines of research suggest that insecure attachment is associated with impairments in self-focused emotional awareness and reflective emotional processing. Research has demonstrated associations between insecure attachment and difficulties in perceiving one’s own emotions, including reduced emotional awareness and increased alexithymia (e.g., [54,55,56]). Likewise, insecure attachment representations have been linked to reduced acceptance of one’s internal experiences and difficulties adopting a non-judgmental attitude toward one’s emotions (e.g., [57,58,59]). These findings suggest that attachment-related differences may affect multiple dimensions of impathy, including the perception, acceptance, reflective observation, and understanding of one’s own emotional states.

Attachment representations have also been linked to *interpersonal emotion perception*. Research demonstrated that secure attachment is associated with enhanced emotion recognition and more adaptive interpersonal emotion processing. For example, Steele et al. [60] showed that secure attachment quality in infancy predicted better emotion recognition abilities in later childhood. Furthermore, studies demonstrated that disorganized attachment is associated with impairments in recognizing and interpreting emotional expressions [61]. In adulthood, attachment security has been further linked to enhanced cognitive empathy and mentalizing abilities. Gallistl et al. [62] demonstrated that adults with secure attachment representations showed higher levels of cognitive empathy compared to insecurely attached individuals. Additional studies reported associations between insecure attachment representations and impairments in interpersonal emotion regulation and social–emotional functioning [63].

Taken together, the available evidence suggests that attachment representations systematically shape both integrated and differentiated forms of emotion perception. However, despite growing empirical interest in these associations, the literature remains fragmented across different conceptual traditions, operationalizations, and methodological approaches. In particular, previous research has rarely differentiated intrapersonal emotion perception according to the multidimensional structure of impathy or systematically distinguished between cognitive and affective facets of empathy. To date, to the best of our knowledge, no systematic review has synthesized the relationship between attachment representations and integrated self–other, intra-, and interpersonal emotion perception within a unified multidimensional framework.

### 1.7. Aim of the Present Review and Hypotheses

The present systematic review aims to synthesize the existing literature on the relationship between adult attachment representations and emotion perception across both intrapersonal and interpersonal domains. Building on the C-FEP framework, the review adopts a multidimensional perspective on emotion perception by integrating research on reflective functioning, impathy, and empathy within a common conceptual framework.

More specifically, the review examines how attachment representations are associated with the four dimensions of intrapersonal emotion perception (impathy)—perceiving, meta-position, accepting attitude, and understanding—as well as with cognitive and affective dimensions of interpersonal emotion perception (empathy). Given the conceptual heterogeneity of the existing literature, the review structures examined constructs of emotion perception into three categories, i.e., *integrated self–other emotion perception* (which is simultaneously related to self- and other-focused emotion perception; e.g., reflective functioning, mentalization), *intrapersonal emotion perception* (Impathy; e.g., alexithymia, interoception), and *interpersonal emotion perception* (Empathy; e.g., emotion recognition, ToM, empathic concern).

Based on attachment theory and the empirical findings outlined above, systematic differences in both intrapersonal and interpersonal emotion perception are expected as a function of attachment representations.

Regarding intrapersonal emotion perception, attachment-related differences are expected to emerge across the four dimensions of Impathy. Secure attachment representations are assumed to be associated with a more coherent and integrated profile, characterized by greater emotional awareness, more differentiated understanding of internal experiences, a more accepting attitude toward emotions, and a more stable reflective meta-position. In contrast, insecure attachment representations are expected to be associated with impairments in emotional self-awareness, emotional understanding, reflective self-related processing, and acceptance-related aspects of emotion perception. Unresolved attachment representations may be associated with broader and more pervasive difficulties across these domains.

Regarding interpersonal emotion perception, secure attachment representations are expected to be associated with higher levels of cognitive empathy, including more accurate mentalizing and emotion-recognition abilities, as well as with more adaptive affective empathy. In contrast, insecure attachment representations are expected to be associated with impairments in cognitive-empathic and mentalizing processes. Unresolved attachment representations may be associated with broader difficulties in the integration of socio-cognitive and socio-affective aspects of interpersonal emotion perception.

Finally, because attachment-related differences are assumed to operate largely outside conscious awareness, the present review focuses on studies that assess attachment at the representational level using narrative-based measures such as the Adult Attachment Interview (AAI) and the Adult Attachment Projective Picture System (AAP). Both measures are designed to “surprise” the unconscious using a semi-structured interview format to assess the individual’s inner working model of attachment.

## 2. Materials and Methods

### 2.1. Search Strategy

The present systematic review was conducted in accordance with the Preferred Reporting Items for Systematic Reviews and Meta-Analyses (PRISMA) guidelines [66]. A structured review protocol and predefined search strategy were developed prior to the literature search; however, the review was not registered in PROSPERO or another systematic review registry, and the protocol was not made publicly accessible. At the time the review was initiated, prospective registration had not yet been established as a standard procedure within our research workflow. Nevertheless, all eligibility criteria, search procedures, and data extraction methods were specified in advance and remained unchanged throughout the review process.

Screening, data extraction, and quality assessment were conducted using DistillerSR (Version 2026.2.1; Evidence Partners, Ottawa, ON, Canada). Both screening phases, as well as data extraction and quality assessment, were performed independently by two reviewers. Disagreements were discussed between the two reviewers until consensus was reached. Because all disagreements could be resolved through consensus between the two reviewers, consultation with a third reviewer was not required.

A systematic literature search was conducted on 9 February 2026, in the electronic databases PsycINFO, PsycArticles, and PubMed. The search strategy was developed using a PEO framework (Population, Exposure, Outcome), as the review focuses on the association between attachment representations and intra- and interpersonal emotion perception rather than on intervention effects. To ensure continuity and comparability with our previous systematic review on attachment and emotion regulation [40], the overall methodological structure was adapted from a previously applied PICO-based framework (Population, Intervention, Comparison, Outcome).

The search syntax included terms related to

(1) Population (adults and adolescents);
(2) Narrative-based attachment representations assessed via the Adult Attachment Interview (AAI) or Adult Attachment Projective Picture System (AAP);
(3) Intra- and interpersonal emotion perception.

Given the conceptual heterogeneity of the literature, the search strategy intentionally included a broad range of constructs related to the dimensions of impathy and empathy. For intrapersonal emotion perception, search terms covered constructs such as interoception, emotional awareness, alexithymia, decentering, cognitive defusion, mindfulness, emotional clarity, and emotion differentiation. For interpersonal emotion perception, the search strategy included constructs related to cognitive and affective empathy, ToM, mentalization, emotion recognition, social cognition, and empathic concern.

The keywords for the search strategy, according to the PEO framework, are presented in Table 1.

In addition to database searches, backward citation chaining of relevant reviews and included articles was conducted on 26 March 2026, to identify additional eligible studies. Manual searches were further performed on the same date to identify potentially relevant studies not captured by the electronic search strategy.

### 2.2. Eligibility Criteria

Eligibility criteria were defined according to the PEO framework. Studies were included if they (a) investigated adolescents or adults, (b) assessed attachment representations using narrative-based attachment measures such as the Adult Attachment Interview (AAI) or Adult Attachment Projective Picture System (AAP), and (c) examined intra- and/or interpersonal emotion perception. The inclusion criteria according to the PEO framework are presented in Table 2.

The following exclusion criteria were applied: (a) studies assessing attachment exclusively via self-report attachment questionnaires, (b) qualitative studies, reviews, theoretical papers, conference abstracts, or case reports, and (c) studies not examining intra- or interpersonal emotion perception.

Studies relying exclusively on self-report attachment measures were excluded because narrative-based attachment interviews are considered more suitable for assessing unconscious attachment-related processes and defensive organization. Given that attachment-related differences in emotion processing are assumed to operate partly outside conscious awareness, the present review specifically focused on representational attachment measures. Importantly, meta-analytic findings suggest only a trivial-to-small overlap between self-report attachment questionnaires and narrative-based attachment interviews, indicating that these approaches capture partially distinct aspects of attachment functioning [67].

### 2.3. Quality Assessment of Included Studies

To assess the methodological quality of the included studies, an adapted version of the Newcastle–Ottawa Scale (NOS) for cohort studies was applied [68]. The scale was modified to fit the objectives of the present review, with attachment representations conceptualized as the exposure variable and intra- and interpersonal emotion perception as outcome variables.

The adapted quality assessment focused on the following domains:

(a) Representativeness of the attachment representation groups;
(b) Selection of comparison groups;
(c) Assessment of attachment representations using validated narrative attachment interviews (AAI or AAP);
(d) Comparability of attachment groups;
(e) Assessment of impathy and/or empathy outcomes.

Each domain was scored according to predefined criteria, resulting in a maximum quality score of six points. Following the structure of the Newcastle–Ottawa Scale, the adapted instrument evaluated methodological quality across the domains of selection, comparability, and outcome assessment.

Quality assessment was performed independently by two reviewers. Disagreements were resolved through discussion and consensus.

### 2.4. Data Synthesis

Given the conceptual and methodological heterogeneity of the included studies, findings were primarily synthesized narratively. Results were organized into three domains:

(1) Integrated self–other emotion perception;
(2) Intrapersonal emotion perception;
(3) Interpersonal emotion perception.

The category of integrated self–other emotion perception was used for constructs that could not be clearly assigned to either intra- or interpersonal emotion perception. Thus, this category refers to processes that are simultaneously related to self- and other-focused emotion perception, particularly reflective functioning.

Within intrapersonal emotion perception, findings were further organized according to the four dimensions of impathy: perceiving, meta-position, accepting attitude, and understanding.

In studies of interpersonal emotion perception, findings were differentiated between cognitive and affective empathy whenever possible.

All relevant results reported in the included studies for these predefined outcome domains were extracted and synthesized narratively. In addition, studies reporting sufficiently comparable effect sizes were included in a quantitative meta-analysis, as described below.

### 2.5. Meta-Analysis

A meta-analysis was conducted to examine the association between attachment representations and reflective functioning. Reflective functioning was selected for quantitative synthesis because it was the only construct assessed with sufficient conceptual and methodological comparability across studies.

Studies were included in the meta-analysis if they assessed attachment using the Adult Attachment Interview (AAI) and reported a direct association with reflective functioning in the form of a correlation coefficient. Attachment was operationalized using AAI-based indicators of attachment security, including coherence of mind, dimensional composite scores derived from AAI state-of-mind ratings, and dichotomous secure–insecure classifications. Dichotomous variables were included when associations were reported as point-biserial correlations, which are mathematically equivalent to Pearson’s r.

When studies reported multiple effect sizes based on independent subsamples, these were aggregated using Fisher’s z transformation and weighted by sample size to obtain a single effect size per study. Effect sizes and corresponding sampling variances were computed as Fisher’s z-transformed correlations using the metafor package in R (RStudio, Version 2026.01.0+392; Posit PBC, Boston, MA, USA).

A random-effects model was applied using restricted maximum likelihood estimation (REML), with Hartung–Knapp adjustment for statistical inference. Effect sizes were back-transformed to Pearson’s r for interpretation. A 95% prediction interval was calculated, and sensitivity analyses were conducted using leave-one-out analyses and influence diagnostics.

Potential reporting bias and small-study effects were assessed using visual inspection of a funnel plot and Egger’s regression test of funnel plot asymmetry. Given the small number of included studies (*k* = 8), the results of these analyses were interpreted with caution. The corresponding funnel plot is provided in the Supplementary Materials (Figure S1).

### 2.6. Use of Generative Artificial Intelligence

Generative artificial intelligence (ChatGPT-5.5, OpenAI, San Francisco, CA, USA) was used in a limited, supportive capacity to assist with language refinement, readability, stylistic improvement, and review of conceptual coherence within the manuscript.

All scientific ideas, theoretical frameworks, hypotheses, methodological decisions, the literature synthesis, interpretations, and conclusions were independently developed, critically evaluated, and authored by the human authors. The authors retained full intellectual responsibility for all content presented in this review.

## 3. Results

### 3.1. Study Selection

**Figure 1:**
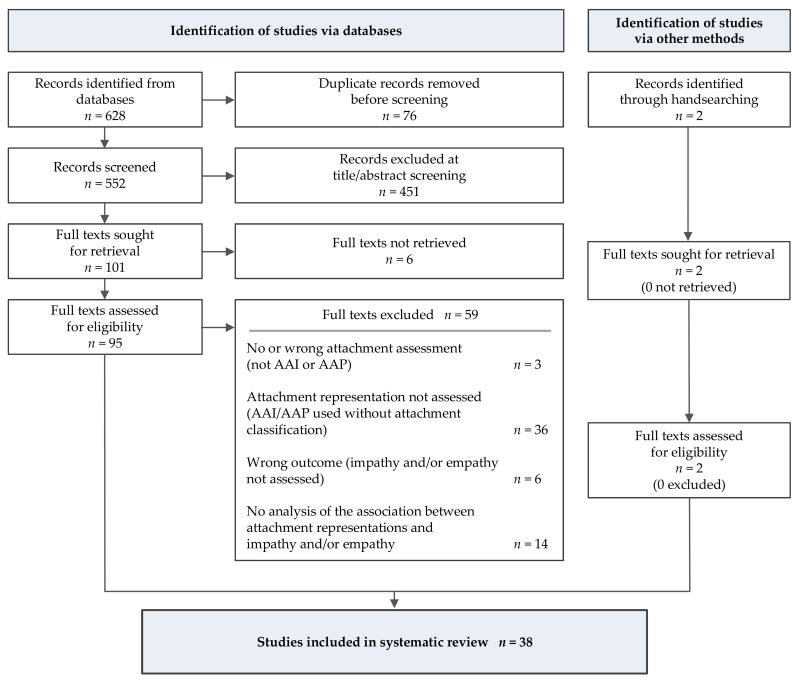
PRISMA flow diagram of the study selection process.

The study selection process is summarized in Figure 1. The database search yielded 628 records, and an additional two records were identified through handsearching. After screening and eligibility assessment, 38 studies met the inclusion criteria and were included in the review. Full-text articles were excluded because of no or wrong attachment assessment (*n* = 3), attachment representation not assessed despite use of AAI/AAP methodology (*n* = 36), no assessment of intra- and/or interpersonal emotion perception (*n* = 6), or no analysis of the association between attachment representations and emotion perception (*n* = 14). Interrater agreement was excellent for both screening phases (κ = 0.92 for title/abstract screening; κ = 0.94 for full-text screening).

### 3.2. Study Characteristics

An overview of the included studies is presented in Table 3. The final sample comprised 38 studies, including a total of 2736 participants from multiple countries and both clinical and non-clinical populations. Most studies were conducted in European countries, particularly Italy, Germany, and Austria, whereas additional studies originated from Canada, the United Kingdom, and the United States. Across studies, sample sizes ranged from 16 to 199 participants.

Attachment representations were predominantly assessed using the Adult Attachment Interview (AAI), which was employed in 32 studies, whereas six studies used the Adult Attachment Projective Picture System (AAP). Regarding population characteristics, 15 studies included clinical samples, 14 investigated healthy samples, and nine included mixed samples comprising both clinical and non-clinical participants.

With regard to emotion perception categories, 23 studies investigated *integrated self–other emotion perception*, primarily through constructs related to reflective functioning and mentalization. Fourteen studies examined *intrapersonal emotion perception (impathy)*, whereas nine studies investigated *interpersonal emotion perception (empathy)*. Several studies addressed more than one emotion perception category simultaneously.

### 3.3. Quality Assessment

Detailed quality ratings for all included studies are presented in Table 4. Overall, the methodological quality of the included studies ranged from low to high, with most studies being rated as moderate quality. Nine studies were classified as high quality, 28 as moderate quality, and one study as low quality. Interrater agreement for the quality assessment was substantial (weighted Cohen’s κ = 0.80). Across studies, quality ratings were generally high for attachment and outcome assessment because all included studies employed validated narrative-based attachment measures (AAI or AAP) and established measures of emotion perception. Lower ratings were primarily attributable to limited statistical control of relevant confounding variables and, in some studies, restricted representativeness of the investigated samples.

### 3.4. Conceptual Mapping of Emotion Perception Measures

The included studies employed a broad range of self-report, observer-based, and performance-based measures assessing partially overlapping aspects of emotion perception. However, these instruments differed substantially regarding the specific emotional processes they captured and the extent to which they reflected integrated self–other emotion perception, intrapersonal emotion perception (Impathy), or interpersonal emotion perception (Empathy).

Within the broader C-FEP framework, which conceptualizes emotion processing as comprising the four core functions of Impathy, Empathy, Emotion Regulation, and Emotion Expression, the present review specifically focuses on the two perceptual core functions: intrapersonal and interpersonal emotion perception. To address the conceptual heterogeneity of the literature within these domains, all included emotion perception measures were systematically mapped onto the multidimensional framework introduced above. Specifically, instruments were classified according to whether they primarily assessed integrated self–other emotion perception, intrapersonal emotion perception, or interpersonal emotion perception. In addition, measures were characterized according to their methodological approach (self-report, observer-based, or performance-based) and the specific emotion perception dimensions they captured.

As shown in Table 5, the included measures varied considerably regarding both methodological approach and conceptual scope. While some instruments selectively assessed circumscribed components of intrapersonal or interpersonal emotion perception, others captured broader reflective or integrative capacities spanning self- and other-related emotional processing. In particular, higher-order Impathy dimensions such as meta-position and accepting attitude were assessed comparatively rarely across studies.

Based on this conceptual mapping and the study categorization presented in Table 3, the findings of the included studies were synthesized according to the emotion perception categories assessed in each study. The following sections, therefore, summarize the evidence separately for integrated self–other emotion perception, intrapersonal emotion perception (Impathy), and interpersonal emotion perception (Empathy), while taking into account the specific assessed emotion perception dimensions and methodological characteristics of the respective measures.

### 3.5. Integrated Self–Other Emotion Perception (Reflective Functioning)

#### 3.5.1. Narrative Synthesis

A total of 23 studies comprising 1664 participants examined integrated self–other emotion perception in relation to adult attachment representations (see Table 3). Most studies assessed integrated self–other emotion perception using the Reflective Functioning Scale (RFS; 22 studies). Of these, 19 studies applied the RFS to Adult Attachment Interview (AAI) narratives, two studies applied the RFS to Adult Attachment Projective Picture System (AAP) narratives, and one study used the RFS only for the Parent Development Interview-Revised (PDI-R). In one AAI-based study, the RFS was also applied to PDI-R narratives. One further study employed the Levels of Emotional Awareness Scale (LEAS), conceptualizing integrated emotion perception as the differentiation and integration of self- and other-related emotional representations.

Several studies consistently demonstrated higher reflective functioning among *securely attached individuals compared to insecurely attached groups*. This pattern was observed across healthy and clinical samples, including psychotherapists in training [86], parents [74,82,83,96,101], adolescents [102], and clinical populations [71,85,88,100,103]. Rosso et al. [96], for example, found that securely attached mothers showed significantly higher reflective functioning than insecurely attached mothers across global reflective functioning and several reflective functioning markers. Maternal reflective functioning was positively associated with coherence of mind and negatively associated with dismissing attachment indicators such as idealization, derogation, and insistence on lack of recall, supporting a close relationship between attachment security and maternal mentalization capacities. In addition, children’s mentalization capacities were positively associated with maternal reflective functioning, particularly mothers’ ability to mentalize negative and mixed-ambivalent mental states. Similarly, Taubner et al. [101] reported that securely attached mothers showed higher reflective functioning, a higher frequency of mind-minded comments—that is, verbal references to the infant’s presumed thoughts, feelings, intentions, or internal states—and lower general and depressive symptom burden compared to insecurely attached mothers. Moreover, the findings tentatively suggested that insecurely attached mothers with lower symptom burden and higher levels of mind-mindedness were more likely to have securely attached infants, pointing to a potential buffering role of maternal mentalization in the intergenerational transmission of attachment insecurity. In depressive patients, Tanzilli et al. [100] demonstrated that securely attached individuals showed significantly higher reflective functioning and more adaptive defensive functioning than insecurely attached or unresolved patients. Furthermore, Jessee et al. [84] found that securely attached individuals showed significantly higher reflective functioning than dismissing individuals, whereas reflective functioning did not significantly differ between secure and preoccupied attachment groups. Similarly, Lenzi et al. [90] reported that securely attached women showed significantly higher reflective functioning than dismissing women, with dismissing participants demonstrating more superficial reflective capacities and less articulated understanding of their own and others’ mental states. Likewise, MacBeth et al. [91] reported impaired reflective functioning among patients with first-episode psychosis characterized by insecure attachment states of mind. Additionally, Tmej et al. [103] found that patients who changed from insecure to secure or from unresolved to resolved attachment classifications during psychotherapy displayed higher reflective functioning both before and after treatment compared to patients whose attachment classifications remained unchanged.

Beyond the general distinction between secure and insecure attachment, several studies highlighted the particular role of *unresolved attachment representations* in impaired reflective functioning and emotional integration. Fischer–Ker, et al. [78] demonstrated that unresolved attachment related to loss was associated with compromised mentalizing capacities in depressive inpatients. Similarly, Subic–Wran, et al. [99], using the LEAS, suggested that unresolved attachment was associated not only with reduced mentalization but also with a lower developmental level of emotional integration, characterized by more implicit and less consciously elaborated emotional processing. Condino et al. [73] further demonstrated that women with organized attachment representations showed significantly higher reflective functioning than women with unresolved attachment representations. Secure attachment was associated with the highest levels of reflective functioning, whereas dismissing and unresolved/cannot classify attachment groups displayed markedly lower reflective functioning scores. Interestingly, higher cumulative trauma exposure was associated with higher reflective functioning, suggesting that mentalization capacities may partly buffer or moderate the psychological impact of traumatic experiences in women exposed to intimate partner violence.

Several studies further conceptualized *reflective functioning as a mediating or developmental mechanism* linking attachment representations to broader psychosocial and clinical outcomes. Taubner et al. [102] demonstrated that reflective functioning partially mediated the relationship between childhood maltreatment and violence potential in adolescence, whereas attachment representations themselves did not emerge as significant mediators. Similarly, Nazzaro et al. [92] reported that reflective functioning mediated the relationship between insecure attachment representations and personality pathology. Studies focusing on parenthood and caregiving further highlighted the developmental significance of mentalization. Dinzinger et al. [74] and Ismair et al. [83] found that parental reflective functioning mediated the relationship between attachment representations and parental sensitivity. Ensink et al. [76,77] further highlighted the relevance of reflective functioning in the context of childhood maltreatment, trauma, and the transition to parenthood. More insecure and unresolved attachment representations were associated with lower levels of reflective functioning toward others, which in turn predicted higher posttraumatic stress symptoms, particularly re-experiencing and hyperarousal symptoms. Reflective functioning partially or fully mediated the association between insecure attachment representations and trauma-related symptom severity, suggesting a potential protective role of mentalization capacities in the context of intergenerational trauma transmission. In addition, securely attached women showed significantly higher levels of general reflective functioning than insecurely attached women, whereas trauma-specific reflective functioning was unrelated to attachment security or unresolved trauma status.

However, two studies found no associations between attachment representations and reflective functioning. Fuchshuber et al. [80], for example, reported that reflective functioning was associated with a richer and more differentiated emotion word repertoire in patients with borderline personality disorder, whereas no significant association emerged between organized attachment representations and reflective functioning. Similarly, Chevalier et al. [72] found no significant associations between maternal insecure attachment (neither preoccupied nor dismissing) and maternal reflective functioning.

Taken together, the available evidence generally suggests that attachment security is associated with more integrated self–other emotion perception (i.e., higher reflective functioning) across both healthy and clinical populations. In contrast, insecure and especially unresolved attachment representations appear linked to impairments in reflective functioning, emotional integration, and self–other processing. Across studies, reflective functioning further emerged not merely as a correlate of attachment security but as a potential developmental mechanism through which attachment representations may influence psychosocial adaptation, psychopathology, parenting, and psychotherapeutic change.

#### 3.5.2. Meta-Analysis

**Figure 2:**
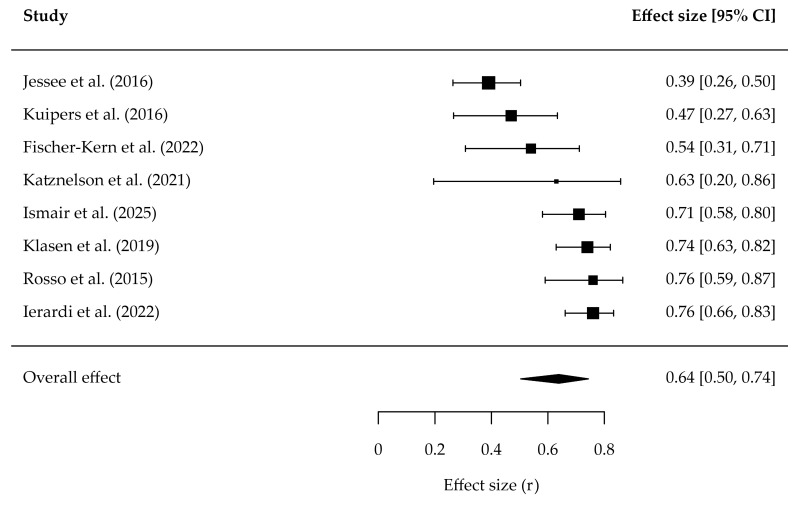
Forest plot of the association between AAI-based attachment security and reflective functioning across included studies. Effect sizes are presented as Pearson’s r with 95% confidence intervals. The diamond represents the overall effect size derived from the REML random-effects model with Hartung–Knapp adjustment. Included studies (from top to bottom in the forest plot) were Jessee et al. [84], Kuipers et al. [88], Fischer-Kern et al. [78], Katznelson et al. [85], Ismair et al. [83], Klasen et al. [86], Rosso et al. [96], and Ierardi et al. [82].

To quantitatively synthesize the association between attachment security and integrated self–other emotion perception, a meta-analysis of studies assessing reflective functioning with sufficiently comparable effect-size data was conducted. A random-effects meta-analysis using REML estimation with Hartung–Knapp adjustment revealed a significant positive association between AAI-based attachment security and reflective functioning, with a large overall effect size (*r* = 0.64, 95% CI [0.50, 0.74]). As illustrated in Figure 2, effect sizes were consistently positive across all included studies (*k* = 8), although their magnitude varied.

Heterogeneity across studies was substantial (*I*^2^ = 77.54%), and the test for heterogeneity was significant (*Q*(7) = 38.58, *p* < 0.001), indicating considerable variability in effect sizes. The 95% prediction interval ranged from *r* = 0.20 to *r* = 0.87, suggesting that the magnitude of the association may vary substantially across comparable future studies.

The included studies comprised both non-clinical and clinical or mixed samples. Given the limited number of studies, sample type was not examined as a separate moderator. However, the inclusion of diverse populations may have contributed to the observed heterogeneity and should be considered when interpreting the findings.

Exploratory moderator analyses examined whether the operationalization of attachment (coherence of mind, composite security measures, or dichotomous classifications) influenced effect size estimates. The overall test of moderators did not reach statistical significance (*p* = 0.083). Composite security measures were associated with smaller effect sizes compared to coherence-based indicators (*p* = 0.035); however, this finding should be interpreted cautiously given the small number of studies and sparse subgroup sizes.

Sensitivity analyses restricted to studies using coherence of mind yielded comparable results, indicating a similarly strong association (*r* = 0.69, 95% CI [0.57, 0.78]). A leave-one-out analysis demonstrated that the overall effect remained stable across all iterations, suggesting that the findings were not driven by any single study. Influence diagnostics identified Jessee et al. [84] as potentially influential. This study reported the smallest effect size while having the largest sample size. Excluding this study reduced heterogeneity and increased the pooled effect estimate, indicating that it did not drive the overall positive association.

Regression-based tests of funnel plot asymmetry were non-significant (*p* = 0.665), although interpretation is limited due to the small number of studies (*k* = 8). Visual inspection of the funnel plot did not indicate pronounced asymmetry; however, interpretation is limited by the small number of included studies. The corresponding funnel plot is provided in the Supplementary Materials (Figure S1).

### 3.6. Intrapersonal Emotion Perception (Impathy)

A total of 14 studies comprising 986 participants examined intrapersonal emotion perception (Impathy) in relation to adult attachment representations (see Table 3). Across studies, intrapersonal emotion perception was operationalized using a broad range of self-report, observer-based, performance-based, and hybrid measures targeting emotional self-awareness, emotional clarity, access to feelings, reflective self-processing, interoceptive perception, alexithymia-related deficits, and differentiated emotional understanding (see Table 5). The most frequently used measure was the Toronto Alexithymia Scale (TAS-20), which was applied in five studies. The Reflective Functioning Scale (RFS) was used in four studies, although this measure primarily captures integrated self–other emotion perception and was therefore considered relevant here only when self-related emotional processing was addressed. Other instruments included the Mental States Rating System (MSRS), Emotion Word Repertoire (EWR), Observer Alexithymia Scale (OAS), Difficulties in Emotion Regulation Scale (DERS), Social Rejection Task, Heartbeat Perception Task, CT-optimal Touch Paradigm, Autonomy-Connectedness Scale-30 (ACS-30), Levels of Personality Functioning Questionnaire (LoPF-Q 12–18), Mayer-Salovey-Caruso Emotional Intelligence Test (MSCEIT), and the Grille de l’Élaboration Verbale de l’Affect (GEVA). Conceptually, the included studies addressed different dimensions of Impathy, particularly emotional self-awareness and access to feelings (*Perceiving*), reflective self-related processing (*Meta-Position*), emotional acceptance (*Accepting Attitude*), and differentiated emotional understanding (*Understanding*). However, many measures captured overlapping aspects of multiple Impathy dimensions rather than isolated components.

Eilert et al. [75], using the Impathy Inventory, directly assessed the four dimensions of intrapersonal emotion perception—Perceiving, Meta-Position, Accepting Attitude, and Understanding—in relation to AAP-based attachment representations in patients with personality disorders and healthy controls. Organized patients showed significantly reduced overall Impathy compared to organized healthy controls despite preserved interpersonal emotion recognition, suggesting that psychopathology was primarily associated with impairments in intrapersonal emotion perception. Specifically, organized patients demonstrated lower scores in Meta-Position, Accepting Attitude, and Understanding. In contrast, unresolved attachment was associated with broader impairments across both intra- and interpersonal emotion processing. Unresolved patients showed the lowest overall Impathy scores and significantly reduced Perceiving compared to organized patients and healthy controls, indicating that difficulties in directly perceiving one’s own emotions may be particularly linked to unresolved attachment-related processes.

Only one study examined more basic forms of bodily and affective self-perception, conceptually related to the Impathy dimension *Perceiving*. Krahé et al. [87], using the Heartbeat Perception Task, found no significant associations between attachment representations and interoceptive accuracy. However, insecure attachment representations were associated with reduced discrimination between CT-optimal and non-CT-optimal affective touch (CT = C-tactile), suggesting differences in the affective perception of affiliative bodily signals.

Across studies, the dimensions *Perceiving* and *Understanding* were most consistently linked to insecure attachment representations. Several studies using the TAS-20 demonstrated elevated alexithymia-related impairments among insecurely attached individuals. Barbasio and Granieri [69], for example, found significantly higher overall alexithymia scores among insecure and unresolved individuals compared to securely attached participants, particularly regarding difficulties describing feelings. Similarly, Taylor et al. [54] reported higher overall alexithymia scores and greater difficulties identifying feelings among insecurely attached women, with alexithymia scores being negatively associated with coherence of mind regarding attachment. Scheidt et al. [98] further demonstrated that dismissing and deactivating attachment strategies were associated with more externally oriented thinking, greater difficulties communicating feelings, and higher overall alexithymia scores, whereas secure attachment was inversely associated with alexithymia scores. Moreover, Gander et al. [81], using the LoPF-Q 12–18, showed that unresolved attachment trauma was associated with impairments in identity functioning and broader self-related personality functioning, including aspects conceptually related to emotional self-awareness and emotional understanding. Similarly, Rosso [95], using the MSCEIT, found that attachment security and reflective functioning were positively associated with emotional intelligence abilities, particularly the capacity to understand emotional states and emotional transitions. In contrast, hyperactivating attachment strategies characterized by anger and passivity were associated with lower emotional intelligence scores related to emotional understanding. Reflective functioning was particularly strongly associated with the MSCEIT emotion-understanding branch.

Additional findings supported associations between insecure attachment representations and reduced emotional awareness and access to internal emotional states. Pace et al. [94], using the DERS, found that insecure attachment representations in adolescents with anorexia nervosa were associated with greater difficulties in emotional awareness and emotional clarity. In particular, maternal idealization and insistence on lack of recall were positively associated with reduced emotional awareness. Similarly, Pace et al. [93] reported that mothers with higher levels of idealization and insistence on lack of recall in the AAI were rated by their daughters as more emotionally uninsightful and alexithymic on the Observer Alexithymia Scale (OAS). Zimmermann [104], using a social rejection task, further demonstrated that secure attachment was associated with greater access to personal feelings, whereas dismissing and deactivating attachment strategies were linked to reduced emotional access in attachment-related distress situations. In contrast, Kuipers et al. [88], using the Self-Awareness subscale of the ACS-30, found no significant association between emotional self-awareness and coherence of mind in patients with eating disorders.

Three studies further linked attachment representations to reflective forms of self-related emotional processing, conceptually related to the Impathy dimension *Meta-Position*. Bouchard et al. [71], using three AAI-based measures of mentalization—the RFS, MSRS, and GEVA—compared reflective functioning, mental states, and verbal affect elaboration in relation to attachment status and psychopathology. Reflective functioning was the only mentalization measure that significantly predicted secure versus insecure attachment status, even after controlling for sex and Axis I or II diagnoses. In contrast, mental-state profiles and verbal affect elaboration did not independently predict attachment security but were more closely related to Axis I and Axis II pathology. Within the Impathy framework, these findings primarily support the relevance of reflective self-related processing and meta-position for attachment security, while affect elaboration appears more strongly linked to psychopathological severity than to attachment status itself. Similarly, Lenzi et al. [90] reported that securely attached women showed significantly higher reflective functioning than dismissing women, whereas dismissing participants demonstrated more superficial reflective capacities and less articulated understanding of their own and others’ mental states. Fuchshuber et al. [80], using the Emotion Word Repertoire, further found that reflective functioning was positively associated with a richer and more differentiated emotional vocabulary in patients with borderline personality disorder, although no direct association emerged between organized attachment representations and reflective functioning.

Evidence specifically addressing the Impathy dimension *Accepting Attitude* was comparatively limited. Only one study examined attachment representations in relation to emotional acceptance-related processes using the DERS [94]. While insecure attachment representations were associated with reduced emotional awareness and emotional clarity, no specific associations emerged for the DERS dimension assessing non-acceptance of emotional responses. Thus, attachment-related differences in the acceptance of one’s own emotional states remain insufficiently investigated and require further empirical study.

Taken together, the available evidence suggests that attachment representations are systematically associated with multiple dimensions of intrapersonal emotion perception (Impathy). Across studies, the most consistent associations emerged for emotional self-awareness, access to feelings, alexithymia-related processes, and differentiated emotional understanding, corresponding primarily to the dimensions *Perceiving* and *Understanding*. In particular, insecure and unresolved attachment representations were repeatedly associated with difficulties identifying, describing, and cognitively processing emotional states, as well as with reduced access to internal emotional experiences. Beyond these alexithymia-related impairments, several studies further linked insecure and unresolved attachment representations—as well as psychopathological functioning—to reduced reflective self-related emotional processing and less differentiated emotional understanding, particularly in relation to reflective functioning and emotional intelligence abilities. The findings of Eilert et al. [75] additionally suggest a differentiated pattern in which psychopathology may primarily affect higher-order reflective and accepting capacities, whereas unresolved attachment appears associated with broader impairments extending to more basic forms of emotional self-perception. In contrast, empirical evidence specifically addressing the Impathy dimension *Accepting Attitude* remained comparatively limited.

Overall, the available findings indicate that insecure and especially unresolved attachment representations are associated with less differentiated, less accessible, and less integrated forms of intrapersonal emotion perception across both healthy and clinical populations.

### 3.7. Interpersonal Emotion Perception (Empathy)

A total of 9 studies comprising 688 participants examined interpersonal emotion perception (Empathy) in relation to adult attachment representations (see Table 3). Across studies, interpersonal emotion perception was assessed using a highly heterogeneous set of performance-based, observer-based, self-report, and hybrid measures targeting different facets of cognitive and affective empathy (see Table 5). Most studies focused primarily on cognitive empathy processes, including Theory of Mind (ToM), perspective-taking, emotion recognition, empathic accuracy, and reflective mentalization capacities related to others’ emotional states. Only one study additionally examined affective empathy directly using the EmpaToM paradigm. Overall, the included studies reflected conceptually diverse research traditions within interpersonal emotion perception rather than a unified operationalization of empathy.

Several studies investigated attachment-related differences in *cognitive empathy* with respect to *Theory of Mind (ToM)* and broader socio-cognitive inference processes. Barone et al. [70], using the Strange Stories Test, demonstrated that securely attached individuals showed significantly better ToM performance than insecurely attached individuals, whereas patients with borderline personality disorder displayed the lowest mentalization abilities. The findings suggested that insecure attachment representations and personality pathology were independently associated with reduced socio-cognitive inferential capacities.

A second group of studies focused on cognitive empathy processes related to *emotion recognition*. Eilert et al. [75], using the Reliable Emotional Action Decoding Test (READ-64-S), examined emotion recognition abilities in relation to AAP-based attachment representations in healthy controls and patients with personality disorders. Organized patients showed preserved interpersonal emotion recognition despite reduced intrapersonal emotion perception, suggesting that psychopathology was more strongly associated with impairments in self-focused than other-focused emotion perception. In contrast, unresolved attachment was associated with broader impairments across both intra- and interpersonal emotion processing. Specifically, unresolved patients demonstrated significantly reduced emotion recognition accuracy compared to organized healthy controls. Similarly, Fizke et al. [79], using a modified Reading the Mind in the Eyes Test (RMET), experimentally investigated the effects of attachment-system activation on emotion recognition performance in healthy individuals and patients with depression. Mentalization was operationalized as cognitive-empathic emotion recognition following attachment-related priming using the Adult Attachment Projective Picture System (AAP). Activation of the attachment system significantly reduced emotion recognition accuracy in individuals with unresolved attachment representations, independent of clinical status. In contrast, securely attached individuals showed stable performance across attachment and neutral conditions. Participants with insecure-organized attachment displayed differential effects depending on psychopathology: healthy individuals with organized insecurity showed enhanced emotion recognition following attachment activation, whereas depressive patients with insecure-organized attachment exhibited reduced performance. Rosso [95], using the Perceiving Emotions branch of the MSCEIT, further found that attachment security and reflective functioning were positively associated with the ability to perceive emotions. In contrast, hyperactivating attachment strategies characterized by anger and passivity were associated with lower scores in emotion perception.

Several studies further examined cognitive empathy processes related to *perspective taking*, *empathic accuracy*, and capacities for *reflective functioning (mentalization)*. Gander et al. [81], using the perspective-taking domain of the LoPF-Q 12–18, demonstrated that unresolved attachment trauma was associated with impairments in perspective taking and broader interpersonal personality functioning in adolescents with childhood trauma histories. Similarly, Sabbagh [97], using the Empathic Accuracy Paradigm during marital conflict interactions, examined the ability to infer a partner’s emotions and thoughts. Securely attached wives showed higher accuracy in inferring their husbands’ emotions than insecurely attached wives, particularly compared to insecure-preoccupied wives. Securely attached wives also demonstrated the highest accuracy in inferring their husbands’ thoughts, whereas preoccupied wives showed the lowest inferential accuracy. Conflict intensity itself was unrelated to empathic accuracy performance. Kungl et al. [89], using the Parental Reflective Functioning Questionnaire (PRFQ), further demonstrated that attachment security was positively associated with parental reflective functioning capacities, particularly regarding interest and curiosity toward the child’s mental states. In contrast, insecure-dismissing attachment representations were associated with reduced parental mentalizing capacities and lower parental sensitivity. Exploratory mediation analyses additionally suggested that reduced parental reflective functioning partially mediated the relationship between dismissing attachment and lower supportive parenting behavior. Thus, the findings indicate that dismissing attachment may be associated with reduced mentalizing and reflective perspective-taking capacities in caregiving contexts.

One study additionally examined *neural correlates of cognitive-empathic processing and mentalizing* in relation to attachment representations. Lenzi et al. [90], using functional magnetic resonance imaging (fMRI), investigated neural responses during empathizing with infant facial expressions in nulliparous women with secure versus insecure-dismissing AAI attachment representations. Within the present framework, the study was conceptualized as involving cognitive empathy processes related to mentalizing and self–other differentiation. Dismissing attachment was associated with increased activation in motor, limbic, and mirror-related brain regions during empathizing tasks, including the inferior frontal gyrus, premotor cortex, superior temporal sulcus, hippocampus, and temporal pole. In contrast, securely attached women showed relatively greater activation in fronto-medial regulatory regions, particularly the perigenual anterior cingulate cortex (pACC) and medial orbitofrontal cortex (mOFC). Dismissing participants additionally showed lower reflective functioning. The authors interpreted this pattern as reflecting heightened emotional reactivity combined with reduced reflective and regulatory capacities during empathic processing. Within the present framework, the findings suggest that dismissing attachment may be associated not with reduced empathic engagement per se, but with less integrated and less reflective forms of cognitive-empathic processing.

Only one study directly addressed *affective empathy*. Gallistl et al. [62], using the EmpaToM paradigm, assessed both cognitive and affective empathy processes. While secure attachment was significantly associated with higher ToM performance, no significant associations emerged between attachment representations and affective empathy or compassion-related processes.

Taken together, the available evidence suggests that attachment representations are systematically associated with multiple dimensions of interpersonal emotion perception (Empathy), particularly cognitive empathy processes. Across studies, the most consistent findings emerged for Theory of Mind (ToM), emotion recognition, perspective taking, empathic accuracy, and reflective mentalization capacities. In general, secure attachment representations were associated with more accurate and differentiated processing of others’ emotional and mental states, whereas insecure and especially unresolved attachment representations were linked to impairments in cognitive empathy-related processes. Several studies further indicated that psychopathology may additionally compromise interpersonal emotion perception, particularly under emotionally activating conditions or in the context of personality pathology. Neurobiological findings further suggest that attachment-related differences in empathy are reflected not only behaviorally but also at the neural level within socio-affective and mentalizing-related brain systems. In contrast, empirical evidence regarding affective empathy remained comparatively limited, with the currently available findings suggesting less robust associations with attachment representations than observed for cognitive empathy processes.

## 4. Discussion

### 4.1. Interpretation of Results

#### 4.1.1. Integrated Self–Other Emotion Perception

Although no separate hypothesis was formulated for integrated self–other emotion perception, this domain was included because reflective functioning serves as a conceptual bridge between intrapersonal and interpersonal emotion perception. Reflective functioning requires individuals to perceive, interpret, and integrate emotional and mental states in both the self and others. The strong positive association between attachment security and reflective functioning observed in the present review and meta-analysis, therefore, extends the hypotheses formulated for impathy and empathy by suggesting that secure attachment is not only associated with more adaptive self- or other-focused emotion perception separately, but also with a more coherent integration of both domains.

This interpretation is consistent with attachment-based mentalization theory, which posits that the capacity to understand mental states develops in early attachment relationships through repeated experiences of contingent affect mirroring and affect co-regulation in caregiver–child interactions [53,64]. Secure attachment may thereby support the development of more coherent reflective capacities by facilitating the integration of affective and cognitive aspects of emotional experience. Conversely, insecure or unresolved attachment may constrain this integration through defensive exclusion, affective hyperactivation, or fragmentation of emotional processing. The present findings therefore further support the view of reflective functioning as a central developmental mechanism linking attachment representations to emotion perception and affect regulation [53,64].

The findings of the present review further align with recent meta-analytic evidence demonstrating that mentalizing capacities are systematically associated with attachment security, psychopathology, affect regulation, personality functioning, and broader psychosocial adaptation across clinical and non-clinical populations [121]. In this sense, reflective functioning may be understood not merely as a narrow socio-cognitive skill but as a broader integrative capacity for organizing emotional experience across self- and other-related domains. This interpretation is also consistent with previous conceptual reviews describing reflective functioning as closely related to self-organization, emotion regulation, and interpersonal functioning [65].

A particularly important implication of the present findings concerns unresolved attachment. Across several included studies, unresolved attachment representations were associated with reduced reflective functioning, lower emotional integration, and impairments in emotionally activating interpersonal situations [73,78,79,99]. These findings fit well with attachment- and trauma-based models proposing that unresolved attachment may compromise the integration of affective, autobiographical, and relational information under conditions of attachment-system activation [53]. Rather than reflecting only a more severe form of organized attachment insecurity, unresolved attachment may therefore represent a qualitatively distinct pattern characterized by disruptions in integrated self–other emotion perception.

At the same time, the present evidence does not yet allow a sufficiently robust differentiation between specific organized-insecure attachment patterns. Although some findings tentatively suggested reduced reflective capacities in dismissing attachment and impairments in emotionally charged inferential processing in preoccupied attachment [90,97], these patterns were not sufficiently consistent across studies to support firm conclusions regarding distinct reflective functioning profiles associated with different organized-insecure attachment strategies. In contrast, unresolved attachment emerged more consistently as a marker of disrupted integration of self–other emotion perception.

Overall, the findings support the view that integrated self–other emotion perception represents a core attachment-related process linking internal working models to broader emotion processing capacities. Within the broader C-FEP framework, reflective functioning may therefore be conceptualized as a higher-order integrative function that coordinates intrapersonal and interpersonal forms of emotion perception, enabling individuals to organize emotional experiences into coherent representations of self, others, and relationships.

#### 4.1.2. Intrapersonal Emotion Perception (Impathy)

The findings of the present review largely support the hypotheses regarding attachment-related differences in intrapersonal emotion perception (Impathy). Across studies, insecure and especially unresolved attachment representations were consistently associated with impairments in emotional self-awareness, alexithymia-related processes, reflective self-related processing, and differentiated emotional understanding. These associations were particularly evident for the Impathy dimensions *Perceiving* and *Understanding*, whereas comparatively less evidence was available for *Meta-Position* and especially *Accepting Attitude*.

Most consistently, insecure attachment representations were associated with difficulties identifying, describing, and cognitively processing one’s own emotional states [54,69,81,93,94,98]. Across several studies, insecure attachment representations were linked to elevated alexithymia scores, reduced emotional awareness, lower emotional clarity, and diminished access to internal emotional experiences. In addition, several findings suggested associations between attachment insecurity and less differentiated forms of reflective self-related processing and emotional understanding [71,75,80,90,95].

The findings further suggest that unresolved attachment may be associated with broader impairments in intrapersonal emotion perception. In particular, Eilert et al. [75] found that unresolved attachment was associated not only with reduced reflective and understanding-related capacities, but also with impairments in directly perceiving one’s own emotional states. Similarly, Gander et al. [81] reported associations between unresolved attachment trauma and impairments in identity functioning and broader self-related personality functioning. However, the available evidence remains comparatively limited, and more research is needed to determine whether unresolved attachment is consistently associated with pervasive impairments across Impathy dimensions.

The present findings are broadly consistent with attachment theory and contemporary models of emotion regulation, which assume that early attachment experiences shape how emotional experiences are perceived, organized, and reflected upon [14,15,16,17,122,123,124]. They further align with meta-analytic findings demonstrating systematic associations between insecure attachment and alexithymia-related impairments [55]. Within this perspective, attachment security may facilitate more differentiated access to emotional experiences and more coherent reflective processing of internal states, whereas insecure attachment representations may be associated with less integrated forms of intrapersonal emotion perception.

At the same time, the present evidence does not yet allow for robust conclusions about distinct intrapersonal emotion-perception profiles associated with specific organized-insecure attachment patterns, such as dismissing versus preoccupied attachment. Although some individual findings tentatively suggested differences in emotional access, emotional understanding, or reflective capacities [90,98,104], these patterns were not sufficiently consistent across studies to support firm conclusions.

Another important finding concerns the comparatively limited evidence regarding the Impathy dimension *Accepting Attitude*. Although emotional acceptance and non-judgmental self-related processing are frequently discussed in contemporary emotion regulation approaches, only one included study directly examined emotional non-acceptance in relation to attachment representations [94], and no specific attachment-related associations emerged. Consequently, the relationship between attachment representations and acceptance-related aspects of intrapersonal emotion perception remains insufficiently understood.

Overall, the findings support the assumption that attachment representations are systematically associated with multiple dimensions of intrapersonal emotion perception. Across studies, insecure and especially unresolved attachment representations were repeatedly linked to less differentiated, less accessible, and less integrated forms of intrapersonal emotion perception across both healthy and clinical populations.

#### 4.1.3. Interpersonal Emotion Perception (Empathy)

The findings of the present review partially support the hypotheses regarding attachment-related differences in interpersonal emotion perception (Empathy). Across studies, secure attachment representations were generally associated with more adaptive forms of cognitive empathy, particularly regarding Theory of Mind (ToM), emotion recognition, perspective-taking, empathic accuracy, and reflective interpersonal processing. In contrast, insecure and especially unresolved attachment representations were associated with impairments in several domains of cognitive empathy. However, the available evidence for affective empathy remained comparatively limited and less consistent.

Most consistently, attachment security was associated with more accurate and differentiated forms of *cognitive empathy*. Across several studies, securely attached individuals demonstrated superior performance in ToM tasks, emotion-recognition paradigms, empathic accuracy, and perspective-taking abilities compared to insecurely attached groups [62,75,79,81,97]. These findings were particularly evident under emotionally activating or attachment-related conditions. For example, Fizke et al. [79] demonstrated that activation of the attachment system selectively impaired emotion-recognition performance in individuals with unresolved attachment representations, whereas securely attached individuals maintained stable performance across conditions. Similarly, Gallistl et al. [62] found that secure attachment was associated with higher ToM abilities, whereas no corresponding associations were observed for affective empathy or compassion.

The reviewed findings further suggest that insecure attachment representations may affect not only the accuracy of interpersonal emotion perception, but also the way emotional information is processed and regulated during empathic interactions. Several studies indicated associations between insecure attachment and less differentiated reflective interpersonal processing, reduced inferential accuracy, or altered empathic processing under emotionally salient conditions [79,90,97]. In particular, the neuroimaging findings reported by Lenzi et al. [90] suggest that dismissing attachment may be associated with heightened emotional reactivity during empathic processing combined with reduced regulatory integration. Within the present framework, these findings tentatively support the assumption that attachment insecurity may involve not simply reduced empathic responding, but less integrated forms of cognitive empathy.

At the same time, the present findings indicate that unresolved attachment may be particularly relevant for understanding impairments in interpersonal emotion perception under emotional arousal. Across several studies, unresolved attachment representations were associated with reduced emotion-recognition accuracy, impaired reflective functioning, and broader disruptions in socio-emotional processing [75,79,81]. These findings are broadly consistent with attachment-based models proposing that attachment-system activation may compromise cognitive empathy by overloading affect-regulation capacities and disrupting reflective processing under emotionally salient conditions [53,64].

In contrast, the evidence regarding *affective empathy* was comparatively sparse and less conclusive. Only one included study simultaneously assessed cognitive and affective empathy processes [62]. Notably, whereas attachment security was associated with superior ToM performance, no significant associations emerged for empathic concern. Thus, the present review provides substantially stronger evidence for attachment-related differences in cognitive empathy than for affective empathy. This imbalance may partly reflect the broader literature, in which cognitive empathy and mentalization processes are more frequently investigated in attachment research than affective empathy.

The present findings are broadly consistent with contemporary social–cognitive and attachment-based models proposing that secure attachment supports more flexible and integrated interpersonal emotion processing [14,15,16,17,20]. They further align with neurocognitive models that distinguish between socio-cognitive and socio-affective empathy systems, suggesting that attachment representations may be particularly relevant to higher-order, reflective, and inferential aspects of empathy rather than to basic affective resonance alone [49,50,51]. Within this perspective, secure attachment may facilitate the capacity to maintain accurate interpersonal understanding even under emotionally activating conditions, whereas insecure or unresolved attachment may interfere with reflective interpersonal processing through defensive regulation strategies, emotional overload, or fragmentation of emotional integration.

Overall, the findings support the assumption that attachment representations are systematically associated with multiple aspects of interpersonal emotion perception, particularly within the domain of cognitive empathy. Across studies, secure attachment was repeatedly associated with more accurate, differentiated, and reflective forms of interpersonal emotion perception, whereas insecure and especially unresolved attachment representations were linked to less integrated and less stable empathic processing across both healthy and clinical populations.

### 4.2. Theoretical Implications for Research of Emotion Processing and Attachment

#### 4.2.1. Toward an Integrative Conceptualization of Empathy

The findings of the present review support the need for a more integrative conceptualization of empathy within attachment and emotion processing research. Across the included studies, interpersonal emotion perception was investigated using a highly heterogeneous set of constructs and operationalizations, including emotion recognition, Theory of Mind (ToM), perspective taking, mentalization, empathic accuracy, reflective functioning, and affective empathy (for a comprehensive overview, see Table 5). Although these constructs originate from distinct yet partially overlapping research traditions, the present findings suggest that they may reflect interconnected functions within a broader system of interpersonal emotion perception.

A comparable multidimensional conceptualization has already been proposed for intrapersonal emotion perception (Impathy). Neubrand [41] conceptualized impathy as a multifaceted process comprising four interdependent dimensions—Perceiving, Meta-Position, Accepting Attitude, and Understanding—which interact dynamically during intrapersonal emotion processing. Building on this process-oriented perspective, the present findings suggest that interpersonal emotion perception (Empathy) may likewise be understood as a multidimensional system that integrates distinct yet interrelated empathic functions, rather than as a unitary construct.

This interpretation is consistent with recent integrative and neuroscientific research on empathy. Contemporary reviews and meta-analyses increasingly suggest that empathy is not a unitary construct but rather a multidimensional process involving perceptual, socio-cognitive, affective, and self–other differentiating components [125,126]. Neuroimaging studies further suggest that affective and socio-cognitive components of empathy rely on partially dissociable neural systems. Affective resonance and affect sharing have consistently been associated with activation in salience-related networks, particularly the anterior insula and anterior cingulate cortex, whereas ToM and perspective taking more strongly recruit temporoparietal and medial prefrontal regions involved in mental state attribution and reflective social cognition [114,126].

Based on the reviewed findings, empathy may be defined as the *capacity to perceive, understand, resonate with, and reflectively differentiate others’ emotional experiences from one’s own*. It may therefore be conceptualized as a multidimensional system of interpersonal emotion perception comprising four interrelated dimensions (see Table 6):

(1) *Perceiving* emotions in others;
(2) *Understanding* others’ emotional and mental states;
(3) *Affective resonance* with others’ emotions;
(4) Maintaining a reflective *meta-position* characterized by self–other differentiation.

Within this framework, the first two dimensions primarily reflect socio-cognitive components of empathy, whereas affective resonance represents the core affective component of empathic processing. In contrast, the meta-position may be understood as a higher-order reflective function enabling adaptive coordination between affective resonance and self–other differentiation.

*Perceiving* refers to the detection and decoding of emotional signals in others, including facial, vocal, bodily, and contextual emotional cues. This dimension is closely related to constructs such as emotion recognition and is supported by meta-analytic evidence demonstrating distinct neurocognitive mechanisms underlying facial emotion recognition [127].

*Understanding* refers to the socio-cognitive interpretation of others’ emotional experiences, intentions, beliefs, and internal states. Constructs such as ToM, perspective taking, mentalization, empathic accuracy, and reflective functioning may be understood as processes contributing to this dimension. Importantly, contemporary evidence suggests that these processes are closely related but not fully identical, reflecting partially distinct mechanisms of interpersonal emotional understanding [126].

*Affective resonance* refers to the capacity to emotionally resonate with another person’s affective state. This dimension corresponds to affective empathy and affective sharing processes described in contemporary empathy research. Experimental paradigms such as the EmpaToM further support the differentiation between socio-cognitive understanding and affective resonance within empathic processing [114].

Finally, the *meta-position* refers to the capacity to maintain reflective self–other differentiation during empathic processing. Importantly, this dimension does not primarily describe emotion regulation itself, which represents a distinct core function of emotion processing within the C-FEP framework, but rather the reflective awareness that affective resonance represents a response to another person’s emotional state rather than one’s own direct emotional experience. This distinction may be particularly relevant for differentiating adaptive empathy from emotional contagion, empathic distress, or interpersonal overinvolvement. Recent neuroscientific evidence further suggests that self–other differentiation relies on partially distinct temporoparietal mechanisms supporting flexible perspective coordination and the differentiation between one’s own and others’ mental and emotional states. Notably, a recent meta-analysis of temporoparietal junction (TPJ) brain-stimulation studies demonstrated causal effects of TPJ modulation on perspective taking, ToM, and self–other distinction, whereas no robust effects were observed for affective empathy, suggesting that TPJ-related mechanisms may primarily support reflective self–other differentiation rather than affective resonance processes [128].

Importantly, compassion should be differentiated conceptually from empathy within this framework. Whereas empathy refers to the perception, understanding, affective resonance, and reflective differentiation of others’ emotional states, compassion primarily describes a prosocial motivational response characterized by warmth, care, concern, and the desire to alleviate suffering. In contrast to affective resonance, compassion does not primarily involve sharing another person’s emotional state but rather reflects an other-oriented motivational response (“feeling for” rather than “feeling with” another person) [129]. Contemporary psychological and neuroscientific findings further suggest that affective resonance, empathic distress, and compassion involve partially dissociable affective and neural processes [129].

The present review further suggests that attachment representations may differentially influence these four dimensions of empathy. Secure attachment was consistently associated with more adaptive interpersonal emotion perception, particularly in relation to emotional understanding, mentalization, and reflective processing of others’ emotional states. In contrast, unresolved attachment representations appeared especially associated with impairments in self–other differentiation and reflective socio-emotional integration under emotionally salient conditions.

This multidimensional perspective may help explain conceptual inconsistencies within previous empathy research. Many studies investigated isolated empathy-related constructs without integrating them within a broader functional framework of interpersonal emotion perception. Consequently, different studies may have assessed distinct empathic functions while using similar overarching terminology. Taken together, the present findings support the view that empathy should not be conceptualized as a unitary construct but rather as a multidimensional system of interpersonal emotion perception integrating perceptual, cognitive, affective, and meta-reflective components.

#### 4.2.2. Emotion Perception and Intergenerational Attachment Transmission

The intergenerational transmission of attachment representations is among the most robust findings in attachment research. Meta-analytic evidence has consistently demonstrated significant associations between caregivers’ attachment representations and children’s attachment security across generations [130,131]. In their large-scale meta-analysis including 95 samples and 4819 parent–child dyads, Verhage et al. [131] confirmed that secure attachment representations in caregivers were significantly associated with secure attachment in children, whereas unresolved attachment representations were associated with increased attachment insecurity and disorganization. Buchheim et al. [132] also demonstrated in a large sample with mothers with childhood maltreatment a significant association of maternal unresolved attachment representation with disorganized attachment in their children. At the same time, attachment research has repeatedly emphasized that the mechanisms underlying this transmission process remain only partially understood.

Classical attachment theory proposed that attachment security develops through caregivers’ capacities to perceive, correctly interpret, and respond appropriately to infants’ emotional and attachment-related signals [133]. Importantly, Ainsworth conceptualized *sensitive responsiveness* not merely as prompt behavioral responding, but as the caregiver’s ability to accurately perceive and meaningfully interpret the child’s internal emotional states. From this perspective, emotion perception processes may represent a central psychological mechanism through which attachment representations influence caregiving behavior and are potentially transmitted across generations.

This assumption is further supported by meta-analytic findings demonstrating that parental sensitivity represents one predictor of attachment security [134]. However, caregiving sensitivity alone does not fully explain intergenerational attachment transmission, resulting in the well-known “transmission gap” described in attachment research [131]. The present findings, therefore, raise the possibility that attachment-related differences in intrapersonal and interpersonal emotion perception may represent additional mechanisms contributing to this transmission process.

The reviewed findings are broadly consistent with this assumption. Across several included studies, secure attachment representations were associated with higher reflective functioning, more adaptive interpersonal emotion perception, greater empathic accuracy, and more differentiated capacities to understand emotional and mental states in both self- and other-related domains [71,74,76,77,83,96,101]. In contrast, insecure and especially unresolved attachment representations were repeatedly linked to impairments in emotional self-processing, reduced reflective functioning, diminished empathic accuracy, and less integrated interpersonal emotion perception [54,69,75,79,81,90,93,94,98]. Importantly, Dinzinger et al. [74] and Ismair et al. [83] further demonstrated that parental reflective functioning mediated the association between attachment representations and parental sensitivity, directly linking attachment-related emotion-perception capacities with caregiving behavior.

Together, these findings suggest that attachment-related differences in emotion perception may influence caregiving sensitivity by shaping how caregivers perceive, mentally represent, and respond to children’s emotional communications.

This interpretation is strongly consistent with research on mind-mindedness and parental mentalization. Meins et al. [135] argued that secure attachment relationships are particularly supported by caregivers’ capacities to accurately perceive and appropriately comment on children’s internal mental states. Importantly, mothers’ appropriate mind-related comments predicted infant attachment security even beyond classical measures of maternal sensitivity. Similarly, Oppenheim et al. [136] demonstrated associations between caregivers’ empathic understanding of children’s internal experiences and children’s attachment security. Taken together, these findings support the assumption that differentiated emotion perception and reflective caregiving capacities may constitute important developmental pathways linking caregivers’ attachment representations with children’s emerging attachment security.

The present review further suggests that both intrapersonal and interpersonal emotion perception may contribute to this process. Difficulties in perceiving and understanding one’s own emotional states may impair affect regulation and reflective caregiving capacities, whereas impairments in interpersonal emotion perception may constrain the accurate interpretation of children’s emotional signals and attachment-related needs. From this perspective, attachment-related impairments in emotion perception may represent one important psychological mechanism underlying insensitive, inconsistent, or emotionally dysregulated caregiving behavior and, consequently, the intergenerational continuity of attachment insecurity.

At the same time, the present review does not allow causal conclusions regarding the precise mechanisms linking attachment representations, emotion perception, and caregiving behavior. Most included studies were cross-sectional and differed substantially in methodology and operationalization. Future longitudinal and experimental research is therefore needed to clarify whether impairments in emotion perception directly contribute to intergenerational attachment transmission and to determine which specific dimensions of emotion perception may be most relevant in this process.

### 4.3. Practical Implications for Psychotherapy, Prevention, and Emotion Coaching

The findings of the present review suggest that both intrapersonal and interpersonal emotion perception may represent important targets for intervention in psychotherapy, prevention, and emotion-focused coaching. Across studies, insecure and especially unresolved attachment representations were associated with impairments in multiple dimensions of intrapersonal emotion perception (Impathy) and interpersonal emotion perception (Empathy). From a clinical perspective, these findings suggest that insecure and especially unresolved attachment representations may be associated not only with impairments in emotion regulation and emotion expression [40] but also with impairments in how emotional states are perceived, understood, and reflectively processed across self- and other-related domains. Together, these findings support the assumption that all four core functions of emotion processing proposed within the C-FEP framework may be systematically associated with attachment representations.

#### 4.3.1. Intervention Targets in Emotion Perception

More specifically, the present findings allow for a differentiated conceptualization of potential intervention targets within both intrapersonal and interpersonal emotion perception. Regarding intrapersonal emotion perception (Impathy), interventions may aim to strengthen the capacity to perceive one’s own emotional states, understand emotional experiences in a more differentiated manner, develop an accepting attitude toward internal affective states, and maintain a reflective meta-position toward one’s own emotions. In relation to interpersonal emotion perception (Empathy), interventions may focus on improving the perception of emotional signals in others, understanding others’ emotional and mental states, fostering adaptive affective resonance with others’ emotions, and maintaining self–other differentiation through reflective meta-position processes.

Importantly, the present findings further suggest that reflective functioning may represent a particularly relevant higher-order capacity linking intrapersonal and interpersonal emotion perception. Within the present framework, reflective functioning may be conceptualized as an integrated form of self–other emotion perception supporting reflective meta-position and self–other differentiation across emotional interactions. Several findings included in the present review support this assumption. Moreover, Tmej et al. [103] demonstrated that higher reflective functioning predicted changes in attachment representations during psychotherapy, suggesting that strengthening reflective capacities may facilitate attachment-related therapeutic change processes. Similarly, Boldrini et al. [137] found that low reflective functioning predicted transition to psychosis in ultra-high-risk individuals, whereas attachment variables alone were less predictive. Kuipers et al. [138] likewise reported that recovery from eating disorders was associated with higher mentalization. Together, these findings suggest that emotion perception, particularly integrated self–other emotion perception, may represent an important target for intervention across different forms of psychopathology.

Recent psychopathology research further underscores the clinical relevance of considering emotion perception in a multidimensional, integrative manner. For example, Pan et al. [139] identified distinct configurations of interpersonal emotion perception in individuals with posttraumatic stress disorder (PTSD) characterized by different patterns across socio-cognitive understanding of others’ emotional and mental states, affective resonance, and self–other differentiation. In particular, a subgroup characterized by reduced perspective-taking capacities and elevated self-focused empathic distress during affective resonance with others showed the highest levels of emotion dysregulation, anxiety, and depressive symptoms. In contrast, individuals with more balanced profiles of interpersonal emotion perception demonstrated comparatively lower psychopathology and better emotional functioning. Importantly, these findings suggest not only that interpersonal emotion perception itself should be conceptualized multidimensionally, but also that impairments in interpersonal emotion perception may dynamically interact with other core functions of emotion processing, particularly emotion regulation. From the perspective of the C-FEP framework, the different core functions of emotion processing may therefore be understood as distinct, yet interconnected systems that dynamically influence and modulate one another across multiple domains of psychological functioning.

The present findings may further help specify intervention targets for different attachment-related emotion-processing patterns. Individuals characterized by affective hyperactivation may particularly benefit from interventions that strengthen reflective meta-position, self–other differentiation, and emotion-regulation capacities to reduce excessive emotional fusion and maladaptive affective overinvolvement. In contrast, individuals characterized by deactivating attachment strategies may benefit from interventions fostering emotional openness, an accepting attitude toward one’s own emotions, and differentiated access to emotional experiences. In this context, compassion-based approaches may help facilitate affiliative motivation and emotional connectedness without requiring excessive emotional overidentification [140]. Overall, the reviewed findings suggest that more individualized and attachment-informed interventions may help tailor therapeutic approaches to different attachment-related emotion-processing patterns.

#### 4.3.2. Implications for Prevention

Beyond psychotherapy and psychopathology, the present findings may also have important implications for developmental prevention approaches. Longitudinal research suggests that early attachment experiences contribute to the development of later interpersonal emotion-processing capacities. For example, Steele et al. [60] demonstrated that early attachment security predicted children’s later emotion-recognition abilities at ages 6 and 11. From this perspective, attachment-related differences in emotion perception may emerge early in development and potentially influence later social, emotional, and psychological functioning across the lifespan.

Consequently, prevention programs may particularly benefit from strengthening caregivers’ capacities for emotion perception, reflective functioning, and sensitive responsiveness during early developmental periods. Supporting parents in perceiving, understanding, and appropriately responding to children’s emotional signals may not only promote attachment security but also foster the development of adaptive intra- and interpersonal emotion-perception capacities. In this sense, attachment-informed prevention approaches may contribute not only to individual emotional development but also to broader long-term mental health promotion.

#### 4.3.3. Implications for Assessment and Process Monitoring

Beyond intervention and prevention, the present findings suggest that intrapersonal and interpersonal emotion perception may also represent valuable targets for clinical assessment and process monitoring.

Assessing emotion-perception capacities may contribute to a more differentiated understanding of individual strengths and vulnerabilities in emotion processing beyond symptom-based assessment alone. Moreover, changes in these capacities across treatment may help identify clinically meaningful developments and provide additional indicators of treatment progress. Such assessment may therefore support a more process-oriented understanding of psychological functioning and may help clinicians identify mechanisms that contribute to adaptive emotional and interpersonal functioning.

### 4.4. Risk of Bias of Included Studies

Several potential sources of bias should be considered when interpreting the findings of the present review. First, most included studies employed cross-sectional designs, limiting causal inferences regarding the directionality of the observed associations between attachment representations and emotion perception. Consequently, it remains unclear to what extent attachment representations shape emotion-perception capacities, whether impairments in emotion perception influence attachment-related functioning, or whether both are reciprocally linked through broader developmental processes.

Second, many studies relied on relatively small and selective samples, particularly within clinical populations. Several investigations focused on highly specific groups, including patients with borderline personality disorder, eating disorders, psychosomatic conditions, or first-episode psychosis. Although these samples provide important insights into attachment-related emotion-processing patterns under conditions of psychopathology, they may limit the generalizability of the findings to broader clinical or non-clinical populations. In addition, some studies included predominantly female samples, potentially limiting conclusions regarding sex-related differences. At the same time, the observation that attachment-related impairments in intra- and interpersonal emotion perception emerged across a wide range of psychopathological conditions may also point toward a broader transdiagnostic relevance of these emotion-perception processes.

Third, substantial conceptual and methodological heterogeneity was evident across studies. Emotion perception was operationalized using a wide range of self-report, observer-based, and performance-based measures originating from partially overlapping conceptual and methodological traditions. While the present review addressed this heterogeneity through a systematic conceptual mapping within the C-FEP framework, differences in operationalization may nevertheless have contributed to variability in findings.

Fourth, comparability between studies was partly limited by inconsistent statistical control of potential confounding variables. Although some studies controlled for factors such as psychopathology severity, age, sex, or educational background, many investigations provided only limited adjustment for relevant covariates. Consequently, some observed associations between attachment representations and emotion perception may partly reflect broader psychopathological, developmental, or social influences.

Finally, conceptual overlap between some attachment-related and emotion-perception-related constructs should be considered. In particular, reflective functioning is closely connected to attachment theory, raising the possibility of partial construct overlap when examining associations between attachment representations and integrated self–other emotion perception. However, the inclusion of diverse methodological approaches and independent performance-based paradigms across studies suggests that the observed associations are unlikely to be fully attributable to shared measurement variance alone.

Despite these limitations, several factors strengthen confidence in the overall findings. All studies employed validated narrative-based attachment measures (AAI or AAP) and established measures of emotion perception. Furthermore, relatively consistent patterns emerged across heterogeneous methodologies, populations, and measurement approaches, particularly regarding the association between secure attachment representations and more adaptive forms of intra- and interpersonal emotion perception.

### 4.5. Future Research Directions

A major direction for future research concerns the need for more longitudinal and developmental approaches to better understand the dynamic relationship between attachment representations and emotion perception across the lifespan. While the present review identified relatively consistent associations between secure attachment representations and more adaptive forms of intra- and interpersonal emotion perception, most available studies relied on cross-sectional designs. Consequently, the developmental pathways underlying these associations remain insufficiently understood. Future longitudinal studies may help clarify whether attachment representations shape the development of emotion-perception capacities over time, whether deficits in emotion perception influence later attachment-related functioning, or whether both processes reciprocally influence one another throughout development. In particular, investigating how early attachment experiences contribute to the emergence of later emotion-processing profiles may further deepen our understanding of transdiagnostic vulnerability and resilience mechanisms.

Future research should also move beyond broad attachment classifications and investigate more differentiated attachment-related defensive and regulatory processes. While the present review identified relatively consistent associations between insecure attachment representations and impairments in intra- and interpersonal emotion perception, the available evidence remains insufficient to clearly differentiate specific emotion-processing profiles across different forms of attachment insecurity. In particular, future studies should more systematically investigate potential differences between dismissing, preoccupied, and unresolved attachment representations in relation to the different core functions of emotion processing. Future research may further examine whether attachment-related defensive processes, such as deactivation, cognitive disconnection, or segregated systems [20], are associated with distinct patterns across these domains (e.g., [75]). Such approaches may contribute to a more nuanced understanding of attachment-related emotion-processing profiles beyond categorical attachment classifications alone.

Another important direction concerns the integration of multimethod and multimodal approaches to emotion-processing research. The present review revealed substantial heterogeneity in the operationalization of emotion perception, ranging from self-report measures to observer-based assessments and experimental paradigms. Future studies may benefit from combining these approaches with psychophysiological, behavioral, and neurobiological methods in order to capture emotion processing more comprehensively across multiple levels of analysis. In particular, narrative-based attachment assessments such as the AAI or AAP may provide promising contexts for examining spontaneous nonverbal expressions of emotion during emotionally salient interpersonal processing. Advances in artificial intelligence and computer vision may further provide valuable tools for the automated analysis of facial expressions (e.g., microexpressions), nonverbal movement behavior, or interpersonal synchrony during attachment-related communication. Such approaches could help bridge attachment research with contemporary affective and social neuroscience while simultaneously increasing the objectivity and ecological validity of emotion-processing assessment.

Finally, the present findings highlight the importance of developing more integrative theoretical models of emotion processing. Across studies, impairments in intrapersonal and interpersonal emotion perception frequently appeared to interact with other core functions of emotion processing, particularly emotion regulation and emotion expression. From the perspective of the C-FEP framework, the different core functions of emotion processing may therefore be conceptualized not as isolated abilities, but as dynamically interacting systems that mutually influence one another across psychological functioning and psychopathology. Future research should further investigate how attachment representations modulate these interactions and whether distinct attachment-related emotion-processing profiles can be identified across different forms of psychopathology. Such approaches may contribute to a more process-oriented and transdiagnostic understanding of mental disorders while also supporting the development of more individualized attachment-informed interventions.

## 5. Conclusions

The present systematic review provides the first integrative synthesis of the relationship between adult attachment representations and intrapersonal and interpersonal emotion perception. Across 38 studies, attachment representations were systematically associated with how individuals perceive, understand, and reflectively process emotional states in themselves and others.

The strongest evidence emerged for reflective functioning, emotional self-awareness, differentiated emotional understanding, and cognitive-empathic processes. Secure attachment representations were consistently associated with more adaptive forms of emotion perception, whereas insecure and especially unresolved attachment representations were linked to less differentiated, less accessible, and less integrated forms of emotional processing. The meta-analysis further demonstrated a large positive association between secure attachment and reflective functioning.

Overall, the findings support the view that attachment representations are systematically associated with the perceptual foundations of emotion processing. In this sense, intrapersonal and interpersonal emotion perception may represent central attachment-sensitive processes contributing to psychological adaptation, psychopathology, caregiving, and therapeutic change.

Future research should employ longitudinal, developmental, multimethod, and integrative approaches to clarify how attachment representations shape the emergence and interaction of different dimensions of intrapersonal and interpersonal emotion perception across the lifespan. In particular, more differentiated investigations of dismissing, preoccupied, and unresolved attachment representations may contribute to a more nuanced understanding of attachment-related emotion-processing profiles.

## Figures and Tables

**Table 1:** Keywords of the search strategy according to the PEO (Population, Exposure, Outcome) approach.

PEO Component	Search Syntax (Keywords)
Population	(adult* OR adolescent*)
Exposure (narrative-based attachment-representation)	(“attachment representation” OR “attachment pattern*” OR “attachment classification*” OR aai OR aap OR “adult attachment interview” OR “adult attachment projective”)
Outcome 1 (impathy)	**Core terms:** impathy OR “introversive empath*” OR “intrapsychic empath*” OR “internal empath*” OR “self-empath*” OR “Impathy Inventory” **Dimensions:** interocept* OR “bodily awareness” OR “emotional awareness” OR “self-awareness” OR alexithymi* OR decenter* OR “self-distancing” OR “cognitive defusion” OR metacognit* OR “meta-position” OR “meta-level” OR accept* OR “self-acceptance” OR mindfulness OR “nonjudgmental” OR “self-understanding” OR “self-knowledge” OR “emotional clarity” OR “emotion* granularity” OR “emotion* differentiation”
Outcome 2 (empathy)	**General concepts:** empathy OR “affective empathy” OR empathiz* OR “cognitive empathy” OR “theory of mind” OR ToM OR “perspective taking” OR “emotion recognition” OR “social perception” OR “social cognition” OR mindreading OR “mind reading” OR “empathic accuracy” OR mentaliz* OR mentalisation **Questionnaires:** “Interpersonal Reactivity Index” OR IRI OR “Empathy Quotient” OR EQ OR “Balanced Emotional Empathy Scale” OR BEES OR “Questionnaire of Cognitive and Affective Empathy” OR QCAE OR “Toronto Empathy Questionnaire” OR TEQ OR „Affective and Cognitive Measure of Empathy” OR ACME OR “Hogan Empathy Scale” OR HES OR “Basic Empathy Scale” OR BES **Performance tests:** “Multifaceted Empathy Test” OR MET OR “Reading the Mind in the Eyes Test” OR RMET OR “Geneva Emotion Recognition Test” OR GERT OR DANVA OR TASIT OR MASC OR BLERT OR PONS OR READ-64

*Note.* The asterisk (*) indicates truncation symbols used in the search syntax to capture multiple word endings and variations. Bold text indicates conceptual categories used to structure the search terms within each PEO component.

**Table 2:** PEO components.

PEO Component	Inclusion Criteria
Population	Adolescents (≥12 years) and adults from clinical and non-clinical population
Exposure	Narrative-based attachment representations assessed via AAI or AAP
Outcome	Constructs related to intrapersonal and/or interpersonal emotion perception

**Table 3:** Overview of included studies (in alphabetical order).

Study	Country	Sample Size	Population	Attachment Assessment	Emotion Perception Category
Barbasio and Granieri [69]	Italy	40	Clinical	AAI	Intrapersonal
Barone et al. [70]	Italy	50	Mixed	AAI	Interpersonal
Bouchard et al. [71]	UK, USA, Canada	73	Mixed	AAI	Integrated/Intrapersonal
Chevalier et al. [72]	Canada	51	Healthy	AAI	Integrated
Condino et al. [73]	Italy	31	Clinical	AAI	Integrated
Dinzinger et al. [74]	Austria	40	Healthy	AAI	Integrated
Eilert et al. [75]	Austria	33	Mixed	AAP	Intrapersonal/Interpersonal
Ensink et al. [76]	Canada	100	Clinical	AAI	Integrated
Ensink et al. [77]	Canada	100	Clinical	AAI	Integrated
Fischer–Kern et al. [78]	Austria	97	Mixed	AAI	Integrated
Fizke et al. [79]	Germany	51	Mixed	AAP	Interpersonal
Fuchshuber et al. [80]	Austria, Germany	87	Clinical	AAI	Integrated/Intrapersonal
Gallistl et al. [62]	Germany	85	Healthy	AAI	Interpersonal
Gander et al. [81]	Austria, Germany	199	Mixed	AAP	Intrapersonal/Interpersonal
Ierardi et al. [82]	Italy	98	Healthy	AAI	Integrated
Ismair et al. [83]	Austria, Germany	80	Healthy	AAI	Integrated
Jessee et al. [84]	USA	194	Healthy	AAI	Integrated
Katznelson et al. [85]	Denmark	16	Clinical	AAI	Integrated
Klasen et al. [86]	Germany	90	Healthy	AAI	Integrated
Krahé et al. [87]	UK	44	Healthy	AAI	Intrapersonal
Kuipers et al. [88]	The Netherlands	71	Mixed	AAI	Integrated/Intrapersonal
Kungl et al. [89]	Germany	115	Healthy	AAI	Interpersonal
Lenzi et al. [90]	Italy	23	Healthy	AAI	Integrated/Intra-/Interpersonal
MacBeth et al. [91]	UK	34	Clinical	AAI	Integrated
Nazzaro et al. [92]	Italy	88	Clinical	AAI	Integrated
Pace et al. [93]	Italy	93	Healthy	AAI	Intrapersonal
Pace et al. [94]	Italy	50	Mixed	AAI	Intrapersonal
Rosso [95]	Italy	93	Healthy	AAI	Intrapersonal/Interpersonal
Rosso et al. [96]	Italy	41	Healthy	AAI	Integrated
Sabbagh [97]	Canada	39	Healthy	AAI	Interpersonal
Scheidt et al. [98]	Germany	40	Clinical	AAI	Intrapersonal
Subic–Wrana et al. [99]	Germany	45	Clinical	AAP	Integrated
Tanzilli et al. [100]	Italy	28	Clinical	AAI	Integrated
Taubner et al. [101]	Germany	53	Healthy	AAP	Integrated
Taubner et al. [102]	Germany	161	Mixed	AAP	Integrated
Taylor et al. [54]	Canada	97	Healthy	AAI	Intrapersonal
Tmej et al. [103]	Austria	63	Clinical	AAI	Integrated
Zimmermann [104]	Germany	43	Healthy	AAI	Intrapersonal

*Note.* AAI = Adult Attachment Interview; AAP = Adult Attachment Projective Picture System. Several studies addressed multiple emotion perception categories simultaneously due to conceptual overlap between integrated self–other, intra-, and interpersonal emotion perception constructs.

**Table 4:** Quality assessment of included studies using the adapted Newcastle–Ottawa Scale.

Study	Selection (0–3)	Comparability (0–2)	Outcome (0–1)	Total Score (0–6)	Quality Rating
Barbasio and Granieri [69]	3	0	1	4	Moderate
Barone et al. [70]	2	0	1	3	Moderate
Bouchard et al. [71]	2	2	1	5	High
Chevalier et al. [72]	2	0	1	3	Moderate
Condino et al. [73]	3	0	1	4	Moderate
Dinzinger et al. [74]	3	0	1	4	Moderate
Eilert et al. [75]	2	2	1	5	High
Ensink et al. [76]	3	0	1	4	Moderate
Ensink et al. [77]	3	0	1	4	Moderate
Fischer–Kern et al. [78]	2	1	1	4	Moderate
Fizke et al. [79]	2	0	1	3	Moderate
Fuchshuber et al. [80]	3	1	1	5	High
Gallistl et al. [62]	3	2	1	6	High
Gander et al. [81]	2	0	1	3	Moderate
Ierardi et al. [82]	3	0	1	4	Moderate
Ismair et al. [83]	3	0	1	4	Moderate
Jessee et al. [84]	3	0	1	4	Moderate
Katznelson et al. [85]	3	0	1	4	Moderate
Klasen et al. [86]	3	1	1	5	High
Krahé et al. [87]	3	1	1	5	High
Kuipers et al. [88]	2	0	1	3	Moderate
Kungl et al. [89]	3	2	1	6	High
Lenzi et al. [90]	2	0	1	3	Moderate
MacBeth et al. [91]	3	0	1	4	Moderate
Nazzaro et al. [92]	3	1	1	5	High
Pace et al. [93]	2	0	1	3	Moderate
Pace et al. [94]	3	0	1	4	Moderate
Rosso [95]	3	1	1	5	High
Rosso et al. [96]	3	0	1	4	Moderate
Sabbagh [97]	3	0	1	4	Moderate
Scheidt et al. [98]	1	0	1	2	Low
Subic–Wran, et al. [99]	3	0	1	4	Moderate
Tanzilli et al. [100]	3	0	1	4	Moderate
Taubner et al. [101]	3	0	1	4	Moderate
Taubner et al. [102]	2	1	1	4	Moderate
Taylor et al. [54]	3	0	1	4	Moderate
Tmej et al. [103]	3	0	1	4	Moderate
Zimmermann [104]	3	0	1	4	Moderate

*Note.* Quality assessment was conducted using an adapted version of the Newcastle–Ottawa Scale (NOS) for cohort studies. Total scores ranged from 0 to 6 points. Scores of 5–6 indicated high quality, scores of 3–4 moderate quality, and scores of 0–2 low quality.

**Table 5:** Conceptual Mapping of Emotion Perception Measures within the C-FEP Framework.

Measure	Method	Primary Classification	Emotion Perception Dimensions Captured	Brief Conceptual Note
Levels of Emotional Awareness Scale (LEAS) [105]	Performance-based	Integrated self–other emotion perception	Impathy: understanding; Empathy: cognitive empathy	Developmental measure of differentiated self- and other-related emotional representations
Reflective Functioning Scale (RFS) [106]	Observer-based	Integrated self–other emotion perception	Impathy: meta-position, understanding; Empathy: cognitive empathy (mentalization)	AAI-based reflective functioning measure integrating self- and other-related mental state processing
Autonomy-Connectedness Scale-30 (ACS-30) [107]	Self-report	Intrapersonal emotion perception	Impathy: perceiving (partial)	Broad self-awareness construct partially overlapping with Impathy
CT-optimal Touch Paradigm [87]	Performance-based	Intrapersonal emotion perception	Impathy: perceiving	Assesses affective perception of affiliative touch
Difficulties in Emotion Regulation Scale (DERS) [108]	Self-report	Intrapersonal emotion perception	Impathy: perceiving, accepting attitude, understanding	Includes non-acceptance of emotional responses
Emotion Word Repertoire (EWR) [109]	Observer-based	Intrapersonal emotion perception	Impathy: understanding	Language-based measure of differentiated emotional vocabulary
Grille de l’Élaboration Verbale de l’Affect (GEVA) [110]	Observer-based	Intrapersonal emotion perception	Impathy: accepting attitude, understanding; Empathy: cognitive empathy (partial)	Assesses affect elaboration and symbolic processing in attachment narratives
Heartbeat Perception Task [111]	Performance-based	Intrapersonal emotion perception	Impathy: perceiving	Measures interoceptive accuracy
Impathy Inventory [41]	Self-report	Intrapersonal emotion perception	Impathy: perceiving, meta-position, accepting attitude, understanding	Multidimensional process-specific Impathy measure
Mental States Rating System (MSRS) [71]	Observer-based	Intrapersonal emotion perception	Impathy: meta-position, understanding	Captures reflective versus defensive modes of mental processing
Observer Alexithymia Scale (OAS) [112]	Observer-based	Intrapersonal emotion perception	Impathy: perceiving, understanding deficits	Observer-rated alexithymia measure
Social Rejection Task [104]	Performance-based	Intrapersonal emotion perception	Impathy: perceiving, understanding	Assesses emotional responses to rejection scenarios
Toronto Alexithymia Scale (TAS-20) [113]	Self-report	Intrapersonal emotion perception	Impathy: perceiving, understanding deficits	Measures alexithymia-related deficits in emotional self-awareness
EmpaToM Task [114]	Performance-based	Interpersonal emotion perception	Empathy: cognitive empathy, affective empathy, empathic concern	Differentiates socio-cognitive and socio-affective processing
Empathic Accuracy Paradigm [115]	Performance-based	Interpersonal emotion perception	Empathy: cognitive empathy, perspective taking	Assesses accuracy of inferred mental states
Parental Reflective Functioning Questionnaire (PRFQ) [116]	Self-report	Interpersonal emotion perception	Empathy: cognitive empathy (parental mentalization)	Assesses mentalization of the child’s internal states
Reliable Emotional Action Decoding Test (READ-64-S) [75]	Performance-based	Interpersonal emotion perception	Empathy: cognitive empathy (emotion recognition)	Facial emotion recognition task
Reading the Mind in the Eyes Test (RMET) [117]	Performance-based	Interpersonal emotion perception	Empathy: cognitive empathy (emotion recognition)	Infers mental states from subtle facial cues
Strange Stories Test [118]	Performance-based	Interpersonal emotion perception	Empathy: cognitive empathy (Theory of Mind)	Advanced social inference task
Levels of Personality Functioning Questionnaire (LoPF-Q 12–18) [119]		Hybrid/ broader construct	Impathy: perceiving, understanding; Empathy: cognitive empathy (perspective taking)	Empathy and self-functioning embedded within broader personality functioning assessment
Mayer–Salovey–Caruso Emotional Intelligence Test (MSCEIT) [120]	Performance-based	Hybrid/ broader construct	Impathy: understanding; Empathy: cognitive empathy (emotion recognition)	Emotional intelligence measure with non-self-referential emotional knowledge

*Note.* Impathy = intrapersonal emotion perception; Empathy = interpersonal emotion perception; AAI = Adult Attachment Interview. Measures were classified according to their primary conceptual focus within the C-FEP (Core Functions of Emotion Processing) framework. Several instruments additionally captured overlapping dimensions of emotion perception.

**Table 6:** Proposed Integrative Dimensions of Empathy.

Proposed Empathy Dimension	Functional Focus	Related Constructs	Measure Represented in the Present Review
Perceiving	Detecting emotional signals in others	Emotion recognition	READ-64-S, RMET, MSCEIT Perceiving Emotions branch
Understanding	Interpreting others’ emotional and mental states	ToM, perspective taking, mentalization, empathic accuracy, reflective functioning	Strange Stories Test, Empathic Accuracy Paradigm, PRFQ, RFS when other-related mentalization is assessed
Affective Resonance	Emotionally resonating with others’ affective states	Affective empathy, affective sharing,	EmpaToM affective empathy component
Meta-Position	Maintaining reflective self–other differentiation during empathic processing	Reflective awareness, self–other distinction	Partially implicated in reflective functioning paradigms

*Note.* The proposed dimensions represent an integrative conceptual framework derived from the heterogeneous constructs assessed across the included studies. The listed measures are intended as illustrative examples of instruments represented in the present review and should not be interpreted as exclusive operationalizations of the respective dimensions. Importantly, the proposed dimensions are conceptualized as functionally interconnected rather than independent processes. While the first two dimensions primarily reflect socio-cognitive components of empathy, affective resonance represents the core affective component of empathic processing, whereas the meta-position is conceptualized as a higher-order reflective function supporting adaptive self–other differentiation during empathic processing. For detailed descriptions of the included measures, see Table 5.

## Data Availability

Materials related to this systematic review, including extracted review data, meta-analytic datasets, and analytic R code, are publicly available via the Open Science Framework (OSF): https://osf.io/xzuqv/ (accessed on 15 June 2026).

## Supplementary Materials

The following supporting information can be downloaded at https://www.mdpi.com/article/10.3390/brainsci16060651/s1, Figure S1 (funnel plot for the meta-analysis on attachment security and reflective functioning); PRISMA 2020 Checklist [141].
